# Activation of FGFR2 Signaling Suppresses BRCA1 and Drives Triple‐Negative Mammary Tumorigenesis That is Sensitive to Immunotherapy

**DOI:** 10.1002/advs.202100974

**Published:** 2021-09-13

**Authors:** Josh Haipeng Lei, Mi‐Hye Lee, Kai Miao, Zebin Huang, Zhicheng Yao, Aiping Zhang, Jun Xu, Ming Zhao, Zenan Huang, Xin Zhang, Si Chen, NG Jiaying, Yuzhao Feng, Fuqiang Xing, Ping Chen, Heng Sun, Qiang Chen, Tingxiu Xiang, Lin Chen, Xiaoling Xu, Chu‐Xia Deng

**Affiliations:** ^1^ Cancer Center Faculty of Health Sciences University of Macau Macau Macau SAR 999078 China; ^2^ Institute of Translational Medicine Faculty of Health Sciences University of Macau Macau Macau SAR 999078 China; ^3^ MOE Frontier Science Centre for Precision Oncology University of Macau Taipa Macau Macau SAR 999078 China; ^4^ Department of Oncology Georgetown‐Lombardi Comprehensive Cancer Center Georgetown University Washington DC 20057 USA; ^5^ Department of General Surgery The Third Affiliated Hospital of Sun Yat‐Sen University Guangzhou Guangdong 510000 China; ^6^ Department of Thyroid and Breast Surgery The Third Affiliated Hospital of Sun Yat‐Sen University Guangzhou Guangdong 510000 China; ^7^ Oncology Laboratory The First Affiliated hospital of Chongqing Medical University Chongqing 400016 China; ^8^ Center of Bone Metabolism and Repair Department of Rehabilitation Medicine State Key Laboratory of Trauma Burns and Combined Injury Trauma Center Research Institute of Surgery Daping Hospital Third Military Medical University Chongqing 400038 China

**Keywords:** BRCA1, breast cancer, FGFR2 inhibitor, FGFR2‐S252W, tumor slice culture

## Abstract

Fibroblast growth factor receptor 2 (FGFR2) is a membrane‐spanning tyrosine kinase that mediates FGF signaling. Various FGFR2 alterations are detected in breast cancer, yet it remains unclear if activation of FGFR2 signaling initiates tumor formation. In an attempt to answer this question, a mouse model berrying an activation mutation of FGFR2 (FGFR2‐S252W) in the mammary gland is generated. It is found that FGF/FGFR2 signaling drives the development of triple‐negative breast cancer accompanied by epithelial‐mesenchymal transition that is regulated by FGFR2‐STAT3 signaling. It is demonstrated that FGFR2 suppresses BRCA1 via the ERK‐YY1 axis and promotes tumor progression. BRCA1 knockout in the mammary gland of the FGFR2‐S252W mice significantly accelerated tumorigenesis. It is also shown that FGFR2 positively regulates PD‐L1 and that a combination of FGFR2 inhibition and immune checkpoint blockade kills cancer cells. These data suggest that the mouse models mimic human breast cancers and can be used to identify actionable therapeutic targets.

## Graphical abstract

In this study, it is demonstrated that activation of FGFR2 promotes triple‐negative breast cancer formation through regulating expression of BRCA1 negatively and PD‐L1 positively. Inhibition of FGFR2, which is widely expressed in human breast cancers, can be combined preclinically with immune checkpoint antibodies to enhance anticancer immunotherapy, warranting clinical evaluation of personalized targeted therapy.

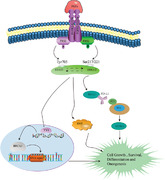

## 1 Introduction

Breast cancers are the most common cancers in women worldwide, and breast cancer‐induced lethality occurs highest among all cancer‐related lethality in females in most countries.^[^ ^1^ ^]^ Approximately 2.3 million newly diagnosed cancer cases and 685 000 deaths were reported by the International Agency for Research on Cancer (IARC) as of December 15, 2020, and ≈10% of all breast cancers are heritable because of germline mutations in breast cancer associated gene 1 (BRCA1), breast cancer associated gene 2 (BRCA2), tumor protein P53 (TP53), ATM serine/threonine kinase (ATM), and others that remain to be identified.^[^ ^2^ ^]^ Easton et al. (2007) analyzed 4398 breast cancer cases and 4316 controls and established that the single nucleotide polymorphisms (SNPs) rs7895676, rs2912781, rs10736303, rs2912778, and rs2981582 in the noncoding region of *FGFR2* were significantly associated with breast cancer.^[^ ^3^ ^]^ Another study reported that the SNPs rs11200014, rs2420946, rs1219648, and rs2981579 in intron 2 of FGFR2 were also associated with breast cancer risk.^[^ ^4^ ^]^ The Fgfr2 fusion genes Fgfr2‐Dnm3 (Dynamin 3), Fgfr2‐Tns1 (Tensin 1), and Fgfr2‐Zmynd8 (zinc finger MYND (Myeloid, Nervy and DEAF‐1)‐type containing 8) were detected in a Brca1‐deficient mouse model.^[^ ^5^ ^]^ Though FGFR2 might promote the progression of mammary tumor, its roles in BRCA1‐associated breast cancer are unknown.

FGFR2 is one of four membrane‐bound receptor tyrosine kinases (RTKs) that mediate the signaling of over 22 fibroblast growth factors (FGFs).^[^ ^6^, ^7^ ^]^ Multiple genetic aberrations in FGFR2 activate upstream and/or downstream FGFR2 signaling pathways and have been identified in breast cancer; 6 out of 165 (3.6%) triple‐negative breast cancers (TNBCs) bear the FGFR2 amplification 10q26.^[^ ^8^ ^]^ Of the 51 screened breast cancer cell lines, MFM223 and SUM52PE manifested *FGFR2* amplification and overexpression. MFM223 and SUM52PE are TNBC cell lines. FGFR2 amplification resulted in activated PI3K‐AKT signaling and inhibition of apoptosis.^[^ ^8^ ^]^ FGFR2 amplification occurs in breast cancer cell lines as well as in normal breast and tumor tissues.^[^ ^9^, ^10^, ^11^ ^]^ It has been reported that 64.8% (81/125) and 56.8% (71/125) of all breast cancers express FGFR2 in the cytoplasm and nucleus, respectively.^[^ ^12^ ^]^ Cytoplasmic FGFR2 expression is significantly associated with tumor size as well as tumor node and metastasis (TNM) stage. Higher cytoplasmic and nuclear FGFR2 levels are associated with low overall survival (OS) and disease‐free survival (DFS) rates than lower FGFR2 levels.^[^ ^12^ ^]^ In contrast, FGFR2 amplification does not affect patient survival.^[^ ^13^ ^]^ Thus, the precise roles of FGFR2 in human breast cancer risk remain to be established.

We previously generated a mouse model for Apert syndrome (AS) bearing FGFR2‐Ser250Trp (S250W) corresponding to human FGFR2‐S252W.^[^ ^14^ ^]^ This mutation occurs in the extracellular domain, enhances ligand‐binding capability, and alters ligand specificity.^[^ ^15^, ^16^ ^]^ The mutant mice presented with severe craniosynostosis characterized by premature coronal suture fusion and shortened cranial bases. It was discovered that the FGFR2‐S252W mutation activates FGFR2‐MAPK‐ERK signaling, which in turn, triggers Bax‐mediated apoptosis.^[^ ^17^ ^]^

In the present study, we showed that FGFR2‐S252W activation promotes TNBC formation by activating the FGFR2/STAT3/MAPK pathway. FGFR2‐S252W negatively regulates BRCA1 via a 39‐bp region in the BRCA1 gene promoter and transcription factor YY1. Moreover, mice bearing Fgfr2 activation display inflammation, PD‐L1 upregulation, and tumor induction. On a tumor slice culture platform, the combination of FGFR2 inhibition plus immune checkpoint demonstrate strong therapeutic efficacy within 4 days.

## 2 Results

### 2.1 Fgfr2 Activation Enhances Mammary Branch Morphogenesis and Promotes Mammary Tumorigenesis

**Figure 1:**
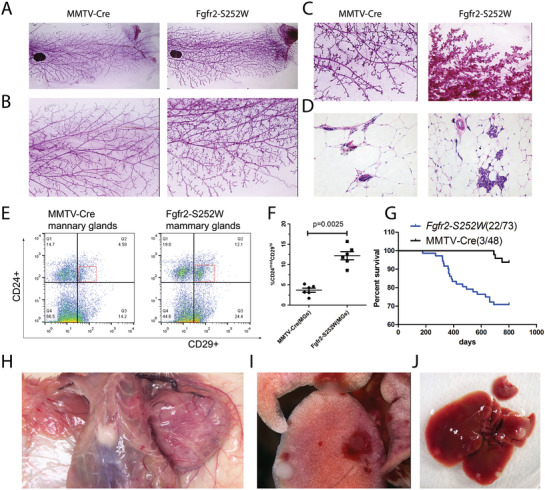
Fgfr2 activation enhances mammary branch morphogenesis and promotes mammary tumorigenesis A–C) Defatted and carmine‐red stained whole‐mount images of the fourth abdominal mammary glands from mice at 3 months A–B) and 6 months C) of ages to indicate genotypes. Three mice were used for each genotype. D) Representative images of mammary glands with Hematoxylin and eosin (H&E) staining of Fgfr2‐WT (*MMTV‐Cre*) and Fgfr2‐S252W. E,F) Representative flow cytometry analysis of the expression of CD24 and CD29 in Fgfr2‐WT, Fgfr2‐S252W mammary glands. Six mice were used for each genotype. G) Percentage of mammary tumor‐free mice as a function of time for *Fgfr2‐S252W* and *MMTV‐Cre*. H–J) *Fgfr2‐S252W* mice develop tumor (H) and metastasis to lung I) and liver J). Data represent the mean ± SEM, *n* = 6. *p* values were determined by an unpaired two‐tailed *t*‐test F). *p* values were carried out using GraphPad Prism 7 Software. *p < 0.05, **p < 0.01, ***p < 0.001, ****p < 0.0001.

To activate Fgfr2 in the mammary tissue, we interbred a mouse strain bearing the *Fgfr2^pLoxpneo‐S252W^* allele^[^ ^14^ ^]^ with another bearing the MMTV‐Cre transgene.^[^ ^18^ ^]^ The progeny were *Fgfr2^pLoxpneo‐S252W^;MMTV‐Cre* (*Fgfr2‐S252W*) mice (Figure S1A,B, Supporting Information). A whole‐mount view of the *Fgfr2‐S252W* mouse mammary glands revealed denser branches and more extensive alveoli than those of wild type (WT) mice. These phenotypic differences were first detected at 2 months and were even more apparent in older animals (**Figure** **1**A–C; and Figure S1C,D, Supporting Information). Histological sections revealed relatively increased cellularity in the hybrid mice (Figure 1D; and Figure S1E, Supporting Information). Flow cytometry of the mammary epithelial cells showed a comparatively increased CD29^Hi^CD24^Med^ population (Figure 1E,F) characteristic of mammary stem cells.^[^ ^19^ ^]^ These observations suggest that Fgfr2 activation enhanced branch morphogenesis and mammary epithelial growth and increased the number of mammary gland stem cells. By 9 months, the *Fgfr2‐S252W* mice gradually developed mammary tumors. 22 out of 73 mice (30%) presented with mammary tumors during the 22 month study period. Three of the control mice (*MMTV‐Cre*) also developed mammary tumors during the same time (Figure 1G). Hence, Fgfr2 activation stochastically induces mammary tumorigenesis after a long latency. Of note, 5 out of 22 mice that developed mammary tumor also carried tumor in the lung, liver, rib cage, or lymph nodes at autopsy (Figure 1H,J; and Figure S1F–G, Supporting Information). Because none of the mammary tumor free mice (*n* = 51) developed these types of tumors, we believed that they were metastasized from the mammary tumors. To confirm this, we have examined these tumors using the mammary epithelial cell marker K14 and found that these tumor cells were K14 positive in the metastatic target organs (Figure S1H–I, Supporting Information).

### 2.2 Fgfr2 Activation Promotes the Development of Triple‐Negative Mammary Tumors

**Figure 2:**
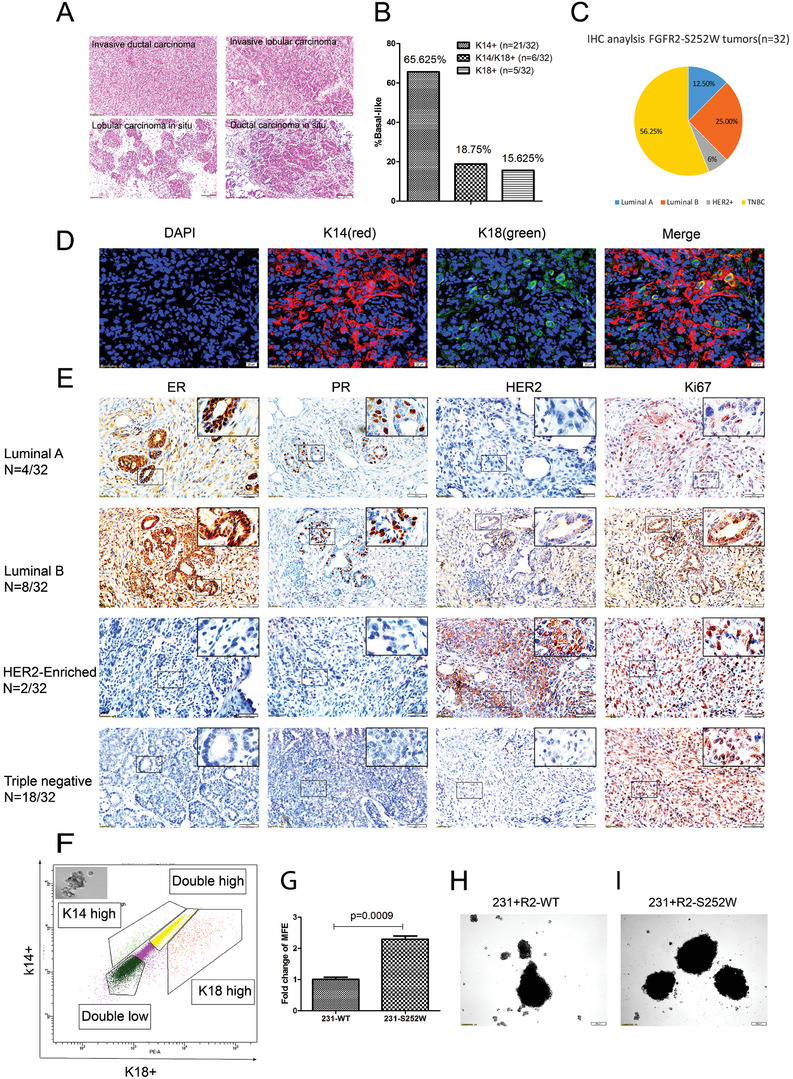
Fgfr2 activation promotes the development of triple‐negative mammary tumors. A) H&E staining (×100) shows the morphologic characters of mammary tumors developed in *Fgfr2‐S252W* mice. Scale bar, 100 µm. B) Summary of K14 and/or K18 incidence in *Fgfr2‐S252W* tumors revealed by Immunofluorescence (IF). C) Summary of incidence of TNBC and other subtypes of tumors revealed by Immunohistochemical (IHC). D) Immunofluorescence (IF) on paraffin sections using K18 (green) and K14 (red) antibodies. DAPI stains the nuclei. E) Immunohistochemical (IHC) staining of ER, PR, HER2, and ki67. F) Representative flow cytometry analysis of the expression of K14 and K18 in *Fgfr2‐S252W* mammary glands. Only K14+ cells could form tumorspheres, which is shown in the insert. G–I)Analysis of the tumor sphere‐forming efficiency (MFE) G). Images of MFE of in MDA‐MB‐231 cells transfected with FGFR2‐WT H) and FGFR2‐S252W I), respectively. Data represent the mean ± SEM and are representative of three independent experiments. *p* values were determined by an unpaired two‐tailed *t*‐test G). Statistical analysis was carried out using GraphPad Prism 7 Software. *p < 0.05, **p < 0.01, ***p < 0.001, ****p < 0.0001.

We characterized the mammary tumors associated with Fgfr2 activation by analyzing the histological features of those that developed in the *Fgfr2‐S252W* mice. 24 out of 32 (75%) of the tumors were invasive ductal carcinoma (IDC), while the remainder comprised invasive lobular carcinoma, lobular carcinoma in situ, and ductal carcinoma in situ (**Figure** **2**A). Immunofluorescence (IF) revealed that 65.625% (21/32) of the mammary tumors were K14+ only. Hence, most of the tumors were basal‐like cancers, 15.625% (5/32) were luminal‐like K18+ only, and 18.75% (6/32) were both K14+ and K18+ (Figure 2B,D) and had mixed K14+ or K18+ cells or K14+/K18+ double‐positive cells (Figure S2A–C, Supporting Information), revealing their transition status. Molecular subtyping revealed that 4/32 (12.5%) of the tumors were the Luminal A type (estrogen receptor‐ and/or progesterone receptor‐positive, HER2‐negative, and low Ki‐67 protein levels), 8/32 (25%) were the Luminal B type (estrogen receptor‐ and/or progesterone receptor‐positive, either HER2‐positive or HER2‐negative, and high Ki‐67 protein levels), 2/32 (6.25%) were the HER2‐enriched type, and 18/32 (56.25%) were TNBCs (Figure 2C,E). Therefore, most of the tumors in the Fgfr2‐S252W mice were TNBCs with high proliferation rates (Figure 2E). A PAM‐50 analysis indicated that 12/18 (66.67%) of the tumors were basal‐like (Figure S2D, Supporting Information).

We then sorted the K14+ only, K18+ only, K14+/K18+, and K14‐/K18‐ cell populations to check tumor sphere‐forming properties. One thousand cells were seeded for clonogenic analysis. Only the K14+ basal‐like cells formed tumor spheres (Figure 2F). Thus, the basal‐like cells may have been more aggressively tumorigenic in this mouse strain. We examined the cancer stem cell (CSC)‐like properties of the tumor sphere‐forming cells by using MDA‐MB‐231‐WT and MDA‐MB‐231‐FGFR2‐S252W cells and found that the latter had a significantly stronger tumor sphere‐forming ability than the former (Figure 2G–I).

### 2.3 Fgfr2 Activation Promotes Epithelial‐Mesenchymal Transition (EMT) Mediated by STAT3‐MAPK Signaling

**Figure 3:**
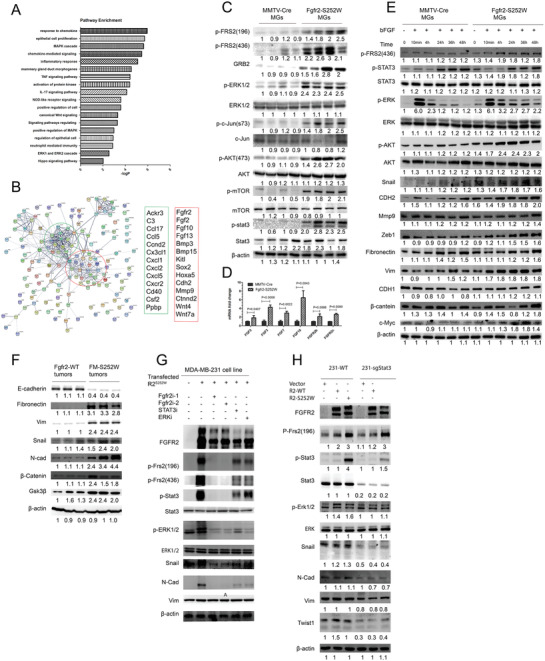
Fgfr2 activation promotes EMT mediated by STAT3‐MAPK signaling. A,B) RNA‐Seq showing differentially expressed genes between *Fgfr2‐WT* and *Fgfr2‐S252W* mammary glands illustrated by pathway enrichment A) and string analysis B). C) Representative Western blot analysis performed on whole‐cell lysates from *Fgfr2‐WT* and *Fgfr2‐S252W* mammary glands to assess the expression of GRB2, FRS2 (Tyr196), FRS2 (Tyr436), ERK1/2 (Thr202/Tyr204), STAT3 (Tyr705), AKT (Ser473), c‐Jun (Ser73), mTOR (Ser2448), and actin. D) Gene profile (FGFs) of Fgfr2‐mediated alterations in the *Fgfr2‐WT* and Fgfr2‐S252W mammary glands analyzed by using real‐time RT‐PCR. Total RNA isolated from 6‐month‐old control and *Fgfr2‐S252W* mice (*n* = 3) was used. E) Fgfr2‐WT and Fgfr2‐S252W mammary gland cells were serum‐starved for 12 h, treated with bFGF for the indicated times, and analyzed for FRS2, ERK1/2, AKT, and EMT markers by WB with antibodies as indicated. F) Representative WB analysis showing expression of EMT marker genes in Fgfr2‐WT and Fgfr2‐S252W mammary gland tumors. G) Representative WB analysis showing alteration of EMT markers with inhibitors to block FGFR2, STAT3, or ERK signaling in MDA‐MB‐231 cells. H) Representative WB analysis showing effects of transfected FGFR2‐WT and FGFR2‐S252W into STAT3 knockout MDA‐MB‐231 cells. Data represent the mean ± SEM and are representative of three independent experiments. *p* values were determined by ANOVA with Tukey's multiple comparison test D). Statistical analysis was carried out using GraphPad Prism 7 Software. *p < 0.05, **p < 0.01, ***p < 0.001, ****p < 0.0001.

We investigated the molecular mechanism by which Fgfr2 activation induces tumorigenesis. We analyzed the transcriptional profiles in the mammary glands of WT and Fgfr2‐S252W mice at 6 months. They were tumor‐free but exhibited enhanced branch morphogenesis. We identified 671 differentially expressed genes (DEGs) between the Fgfr2‐S252W and WT mammary glands (gene list in Excel 1, Supporting Information). Of these, 359 were upregulated and 312 were downregulated. Metascape analysis^[^ ^20^ ^]^revealed pathways related to epithelial cell differentiation, proliferation, inflammatory response, MAPK cascade, chemokines, and so on (**Figure** **3**A). A sub‐PPI network was reconstructed as shown in Figure 3B. Two groups of genes were positively linked to inflammatory factors (green box) and growth factors/EMT (red box), respectively. Gene Set Enrichment Analysis (GSEA) revealed high activation of breast cancer pathway (Figure S3A, Supporting Information) and MAPK (Figure S3B, Supporting Information) signaling in the *Fgfr2‐S252W* mammary glands compared with those of *MMTV‐Cre*. Western blot analysis of multiple genes in the MAPK/ERK, PI3K/AKT/mTOR, and JNK/c‐JUN pathways showed markedly upregulated FRS2*α*, GRB2, ERK1/2, c‐JUN, AKT (Ser473), mTOR, and STAT3 phosphorylation (Figure 3C). Therefore, some of these might have been responsible for tumor formation.

RNA‐seq displayed significantly upregulated Fgf2 and Fgf10 in the mutant mammary glands and two more Fgfs: Fgf3 and Fgf7 in the tumors (Figure 3B,D). It was shown that FGFR2‐S252W enhances its ligand‐binding capability.^[^ ^15^, ^16^ ^]^ Consistently, our co‐immunoprecipitation (co‐IP) study revealed markedly increased interaction between FGFR2‐S252W and FGF3 and FGF7 (Figure S3C–D, Supporting Information). Thus, we hypothesized that while Fgfr2‐S252W partially activates FGFR2 signaling, the increased FGF ligand expression could further enhance it. We subjected Fgfr2‐S252W and WT mammary epithelial cells to FGF2 (bFGF) and checked downstream signaling including FRS2*α*, ERK1/2, AKT, EMT, and so on. MAPK‐ERK signaling rapidly responded and gradually disappeared after 2 h. In contrast, STAT3 signaling was activated 24 h later and sustained thereafter (Figure 3E). There were also higher levels of the EMT proteins Snail, CDH2, MMP9, and Vim in Fgfr2‐S252W than WT mammary epithelial cells. Hence, FGF ligands could further enhance Fgfr2‐S252W‐mediated signaling and play important roles in Fgfr2‐S252W mammary tumorigenesis.

Expression of the epithelial cell marker E‐cadherin was lower in Fgfr2‐S252W mammary tumors than that in Fgfr2 WT mammary tumors. However, immunofluorescence (IF) confirmed the opposite trends for the mesenchymal markers: fibronectin, *N*‐cadherin, vimentin, and Snail (Figure 3F; and Figure S3E, Supporting Information). We then assessed the effects of inhibiting STAT3 and ERK on EMT induction by FGFR2‐S252W signaling in MDA‐MB‐231 cells. The levels of pFRS2(Tyr196), pSTAT3(Tyr705), pERK1/2, *N*‐Cad, and Snail (Figure 3G) markedly increased upon FGFR2‐S252W expression. However, these responses were completely reversed upon inhibiting Fgfr2 with BGJ398 or AZD4547. STAT3 inhibition with C188‐9 significantly inhibited pERK1/2, *N*‐Cad, and Snail. ERK inhibition with U0126 had milder effects on the pSTAT3 and Snail levels but its impact on *N*‐Cad resembled that of STAT3i. These data suggest that STAT3 is the main mediator of Fgfr2 signaling. To further investigate this, we used the CRISPR/Cas9 system to knock out STAT3 in MDA‐MB‐231 cells and then transfected Fgfr2‐WT and Fgfr2‐S252W into the knockout cell lines. Upregulation of these proteins by Fgfr2 was significantly suppressed in the STAT3 knockout (Figure 3H; and Figure S3F, Supporting Information), confirming STAT3 is the main mediator of Fgfr2 signaling in ERK activation and regulates EMT gene expression.

### 2.4 FGFR2 Activation Negatively Regulates BRCA1 by Suppressing Transcription Factor YY1 Mediated by the FRS2*α*/STAT3/MAPK Pathways

**Figure 4:**
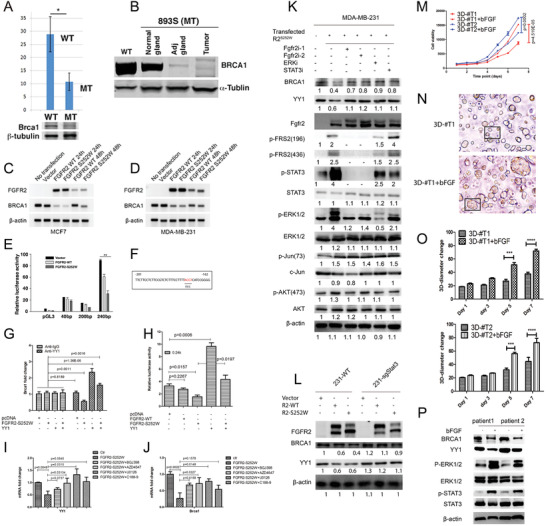
FGFR2 activation negatively regulates BRCA1 by suppressing transcription factor YY1 mediated by the FRS2*α*/STAT3/MAPK pathways. A,B) Expression of Brca1 revealed by RT‐PCR and WB in *Fgfr2‐WT* and *Fgfr2‐S252W* tumors. C,D) BRCA1 mRNA level expression after transfected FGFR2‐WT and FGFR2‐S252W in MDA‐MB‐231 and MCF7 cells revealed by RT‐PCR. E) Luciferase activities of BRCA1 reporter constructs in MDA‐MB‐231 cells. Fold change in luciferase activity of the BRCA1 promoter after transfection of FGFR2‐WT and FGFR2‐S252W. F) Transfection factor YY1 binding site. G‐H) ChIP assay showing that FGFR2‐S252W can compete with YY1 binds to the BRCA1 promoter in MDA‐MB‐231 cells transiently transfected FGFR2‐S252W and co‐transfected with YY1. I,J) FGFR2 mainly though FGFR2/FRS2/MAPK signaling to regulate YY1 and BRCA1. The functionality of FGFR2 in MDA‐MB‐231 cells as revealed by transfection of FGFR2 overexpression vector or empty vector for 24 h, then treated with multiple inhibitors for another 24 h. Expression of YY1(I) and BRCA1(J) were measured by RT‐PCR. K) FGFR2 suppress BRCA1 and YY1 were rescued by inhibit FGFR/FRS2/STAT3/MAPK signaling evaluated by immunoblotting. Actin was used as a loading control. L) Immunoblotting assay evaluating the expression of YY1/BRCA1 in STAT3‐deficiant cells after transfecting with FGFR2‐WT and FGFR2‐S252W. M–P) Growth curves of two organoid lines (3D‐#T1 and 3D‐#T2) with/without bFGF treatment measured by MTT continuously up to 7 days M), sizes of the organoids N,O) measured by using image J, and expression of some downstream signaling revealed by western blot (P). Data represent the mean ± SEM and are representative of three independent experiments. *p* values were determined by ANOVA with Tukey's multiple comparison test E, G, H, I, J, M, and O). Statistical analysis was carried out using GraphPad Prism 7 Software. *p < 0.05, **p < 0.01, ***p < 0.001, ****p < 0.0001.

Most of the tumors that developed in Fgfr2‐S252W were BL‐like and TNBCs. Hence, we examined the expression of Brca1 because its deficiency triggers BL‐like and TNBC cancer formation.^[^ ^21^, ^22^ ^]^ RT‐PCR revealed that the Brca1 mRNA levels were lower in mutant mammary glands and tumors than in the controls (**Figure** **4**A). Western blotting revealed graded reductions in the levels of Brca1 in mutant glands, adjacent tissue, and tumors compared with the Brca1 levels in WT glands (Figure 4B). Gene Set Enrichment Analysis (GSEA) revealed downregulated Brca1 signaling (Figure S4A, Supporting Information) in Fgfr2‐S252W compared to that in Fgfr2‐WT mammary glands. Therefore, FGFR2 signaling downregulates BRCA1 transcription. We transfected MCF7 and MDA‐MB‐231 cells with constructs expressing human FGFR2 and FGFR2‐S252W and found that ectopic FGFR2, especially FGFR2‐S252W expression, decreased BRCA1 mRNA level (Figure 4C,D). Our previous study indicated that BRCA1 transcription is both positively and negatively regulated.^[^ ^23^ ^]^ BRCA1 downregulation in mutant mice may suggest that FGFR2 signaling negatively regulates BRCA1 transcription. We constructed a luciferase reporter series encompassing a 1704 kb regulatory region of the human BRCA1 reporter (between −1460 and +244 bp) (Figure S4B, Supporting Information). All reporter constructs containing the *β* promoter (type I vector) or lacking the *β* promoter and containing the region beyond −201 bp (type II vector) exhibited significantly lower luciferase activity than the 0.24 kb construct (type III vector) containing the *α* promoter (Figure S4C, Supporting Information). Therefore, the *β* promoter and the region beyond −201 bp negatively regulate BRCA1 transcription, whereas the 0.24 kb fragment positively regulates BRCA1 expression. We created a serial deletion construct (type IV vector) by gradually truncating about 40 bp distant from the 0.24 kb region. A region between −201 and −162 bp (39 bp) maintains luciferase activity because all type IV constructs lacking this region exhibited far lower luciferase activity. However, a construct containing this 39‐basepair alone (type V vector) also showed low luciferase activity (Figure S4C, Supporting Information). Hence, other regions in the 0.24 kb fragment might also be required for the positive regulation of BRCA1 transcription.

We then cotransfected FGFR2‐WT or FGFR2‐S252W to investigate whether FGFR2 signaling inhibits luciferase activity in the 0.24 kb reporter. A graded reduction in BRCA1 expression was observed from the FGFR2 to the FGFR2‐S252W construct (Figure 4E). We had previously indicated that YY1 positively regulates BRCA1 expression by binding a YY1 consensus site in a 39‐basepair region between −162 and −201 bp (Figure 4F).^[^ ^23^ ^]^ We found that in FGFR2 mutant mice, YY1 expression was reduced at the transcriptional and translational levels and in both mammary tissues and tumors. This finding correlated with the markedly reduced BRCA1 levels (Figure S4D–H, Supporting Information). We also have analyzed 60 breast cancer samples by immunohistochemistry (IHC) and found FGFR2 is negatively correlated with YY1 and BRCA1 (Figure S4I, Supporting Information), suggesting that YY1 could be responsible for BRCA1 reduction in response to FGFR2 activation.

Next, we used a chromatin immunoprecipitation (ChIP) assay to investigate whether FGFR2 prevents YY1 binding to the BRCA1 promoter. FGFR2‐S252W expression blocked YY1 binding to the BRCA1 promoter (Figure 4G). YY1‐activated BRCA1 Luc reporter transcription was also blocked (Figure 4H). To clarify the molecular mechanism by which FGFR2‐S252W regulates BRCA1, we transfected FGFR2‐S252W into five different breast cancer cell lines. Western blotting demonstrated that FGFR2‐S252W reduced YY1 and BRCA1 expression in four of the cell lines. However, the MDA‐MB‐436 cell line had low YY1 and high BRCA1 levels. Therefore, BRCA1 expression in MDA‐MB‐436 may have been independent of YY1 (Figure S4J, Supporting Information).

We reported that YY1 positively regulates BRCA1.^[^ ^23^ ^]^ Thus, we hypothesized that FGFR2‐S252W suppresses BRCA1 via YY1. We transfected FGFR2‐S252W into MDA‐MB‐231 cells and found it significantly reduced levels of mRNA (Figure 4I,J) and protein (Figure 4K) of both YY1 and BRCA1, and such inhibition effects could be largely overridden by inhibitors for FGFR (BGJ398 and AZD4547), STAT3 (C188‐9), or ERK (U0126) (Figure 4I–K). These data suggest that the regulation of YY1/BRCA1 by FGFR2 might go through STAT3 and ERK/MAPK signaling. Consistent with the notion that STAT3 represses BRCA1 and YY1 expression. STAT3 knockout in MDA‐MB‐231 cells restored the YY1 and BRCA1 protein levels that had been suppressed by FGFR2‐S252W (Figure 4L). To investigate this further, we used two breast patient derived organoids (PDOs) that we have previously generated^[^ ^24^ ^]^ and treated them with bFGF (50 ng mL^−1^). The data showed that the treatment increased cell proliferation (Figure 4M) and organoid sizes (Figure 4N,O; and Figure S4K, Supporting Information), while did not have obvious effect on cell morphology. Besides, the data also showed significantly reduced BRCA1 and YY1 and activated STAT3 and MAPK (Figure 4P). Altogether, it is evidence that FGFR2 regulates BRCA1 through YY1 in mammary tissues and tumors and this process is mediated by STAT/ERK signaling.

### 2.5 Cooperation between Fgfr2 Activation and Brca1 Deficiency Accelerates Mammary Tumorigenesis

**Figure 5:**
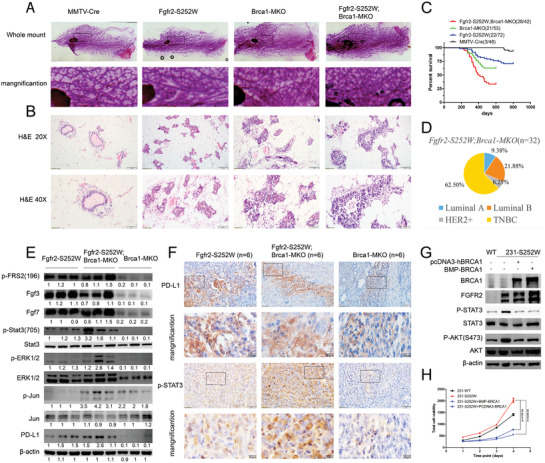
Cooperation between Fgfr2 activation and Brca1 deficiency accelerates mammary tumorigenesis. A,B) Defatted and carmine‐red stained whole‐mount mammary glands A) and H&E staining B) with indicated genotype. C) Percentage of mammary tumor‐free mice with indicated genotype (*n* = number of mice). D) Percentage of mammary tumors molecule subtypes with indicated genotype (*n* = number of tumor). E) Representative WB analysis performed on whole‐cell lysates from tumors indicated. F) IHC staining against PD‐L1 and pSTAT3 in tumors with genotype indicated. G) Western blots using various antibodies for the effect of ectopic expression of BRCA1 on 231‐S252W cells. H) Two BRCA1 expression plasmids were transfected into 231‐WT and 231‐S252W cells. 24 h later, 3000 cells were seeded into each well of 96‐wells plate. Cells were monitored for four consecutive days and cell proliferation measured and quantified by Alamar blue. Data represent the mean ± SEM and are representative of three independent experiments. *p* values were determined by ANOVA with Tukey's multiple comparison test (H). Statistical analysis was carried out using GraphPad Prism 7 Software. *p < 0.05, **p < 0.01, ***p < 0.001, ****p < 0.0001.

Reduced BRCA1 expression in *Fgfr2‐S252W* mice suggests that BRCA1 is implicated in tumorigenesis. As BRCA1 suppresses tumor growth, we hypothesized that full BRCA1 dosage might inhibit tumorigenesis, while BRCA1 suppression by FGFR2 signaling could weaken this inhibition. If this theory is valid, then tumorigenesis in Fgfr2‐S252W mice should be enhanced by reducing BRCA1. We crossed the *Fgfr2‐S252W* mice with those bearing the mammary gland‐specific Brca1 knockout (*Brca1^Co/Co^;MMTV‐Cre*, or *Brca1‐MKO*)^[^ ^25^ ^]^ and generated the double mutant *Fgfr2‐S252W;Brca1‐MKO*. Relatively more extensive branch morphogenesis is observed in the mammary glands of the *Fgfr2‐S252W;Brca1‐MKO* mice than that observed in each parental line bearing a single mutation (**Figure** **5**A,B). At about 6 months, the *Fgfr2‐S252W;Brca1‐MKO* mice began to develop mammary tumors. The median time was 10 months. In contrast, the *Fgfr2‐S252W* and *Brca1‐MKO* mice manifested mammary tumors at median times of 15 and 21 months, respectively (Figure 5C). Therefore, tumorigenesis in *Fgfr2‐S252W;Brca1‐MKO* mice occurred significantly faster than in each parental line bearing a single gene mutation. The incidence of TNBC in the *Fgfr2‐S252W;Brca1‐MKO* mice was 62.5% (19/32). (Figure 5D). Hence, while Fgfr2 activation and Brca1 deficiency markedly enhanced mammary tumorigenesis, they also increased TNBC formation.

To elucidate accelerated tumorigenesis, we evaluated the expression of certain genes associated with FGFR2 signaling in tumors excised from *Fgfr2‐S252W*, *Fgfr2‐S252W;Brca1‐MKO*, and *Brca1‐MKO* mice. Tumors from the *Fgfr2‐S252W;Brca1‐MKO* mutant mice manifested elevated FGF3, FGF7, and pFRS2 levels as did those from the *Fgfr2‐S252W* mice. Tumors from the double mutant mice also displayed higher levels of pSTAT3, pERK1/2, and p‐cJun than the *FGFR‐S252W* and *Brca1‐MKO* tumors (Figure 5E,F). The data suggest that BRCA1 might retard cell growth through inhibiting these FGFR2 signaling pathways. To further investigate this, we ectopically expressed BRCA1 in 231‐S252W cells and found it indeed suppressed the enhanced pSTAT3 and pAKT triggered by FGFR2‐S252W (Figure 5G), and also significantly inhibited proliferation of both MDA‐MB‐231 and 231‐S252W cells (Figure 5H). Altogether, these data suggest that double‐mutant tumors activate more oncogenic signaling than single‐mutant tumors, accounting for the accelerated tumorigenesis. Of note, IHC revealed comparatively higher levels of the immune checkpoint protein PD‐L1 in the *Fgfr2‐S252W* tumors. PD‐L1 upregulation was greater in the *FGFR‐S252W;Brca1‐MKO* double mutant tumors (Figure 5F).

### 2.6 PD‐L1 Expression is Promoted by FGFR2‐Induced STAT3 and ERK Activation

**Figure 6:**
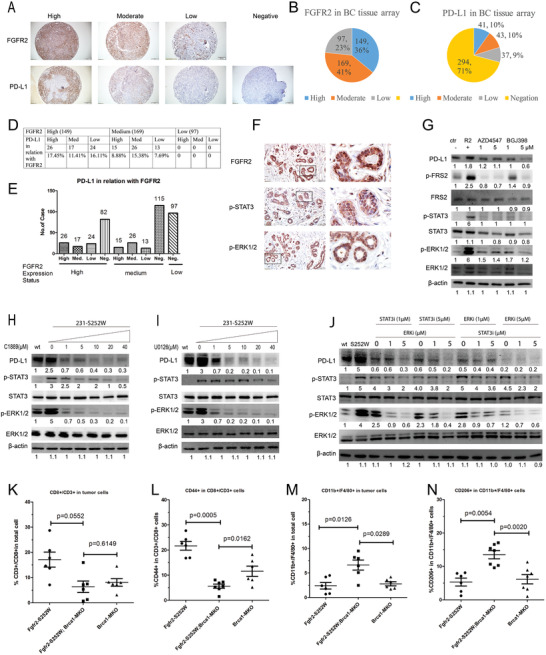
PD‐L1 expression is promoted by FGFR2‐induced STAT3 and ERK activation. A–F) Expression of FGFR2 in human breast cancer tissue array and co‐expression of PD‐L1, p‐STAT3, and p‐ERK1/2. G) PD‐L1 was assessed by WB after being treated with FGFR inhibitors for 24 h in MDA‐MB‐231 cells and FGFR2‐S252W transfected FGFR2‐S252W cells. H–I) PD‐L1 was assessed by WB after being treated with different concentrations of C1889 and U0126 for 24 h in FGFR2‐S252W cells. J) FGFR2 activation promotes expression of PD‐L1 through STAT3‐ERK signaling. K,L) CD8+ T cells and activated CD8+ T cells in Fgfr2‐S252W, Brca1‐MKO, and Fgfr2‐S252W;Brca1‐MKO tumors analyzed by flow cytometry. M‐N) M1/M2 macrophage (F4/80, CD11b, and CD206) cells in Fgfr2‐S252W, Brca1‐MKO and Fgfr2‐S252W; Brca1‐MKO tumors analyzed by flow cytometry. Data represent the mean ± SEM and are representative of three independent experiments. *p* values were determined by ANOVA with Tukey's multiple comparison test J, K, L, and M). Statistical analysis was carried out using GraphPad Prism 7 Software. *p < 0.05, **p < 0.01, ***p < 0.001, ****p < 0.0001.

Next, we investigated the expression of FGFR2 and PD‐L1 using IHC in a tissue array comprising 415 human breast cancer samples. All samples expressed FGFR2 at measurable levels. Among these, 149 (149/415; 36%), 169 (169/415; 41%), and 97 (97/415; 23%) expressed FGFR2 at high, medium, and low levels, respectively (**Figure** **6**A,B). PD‐L1 was found at detectable levels in 121 samples (121/415; 29%). Of these, 41 (41/415; 9.87%), 43 (43/415; 10.36%), and 36 (37/415; 8.92%) expressed PD‐L1 at high, medium, and low levels, respectively. The remaining 294 samples (294/415; 70.84%) were negative for FGFR2 and PD‐L1 (Figure 6A,C). To clarify the relationship between FGFR2 and PD‐L1, we compared their expression in each sample using IHC. FGFR2 expression was positively correlated with PD‐L1 expression (Figure 6D,E). Taken together, these data demonstrate that FGFR2 activation is positively associated with PD‐L1 expression in breast cancer. Furthermore, we also checked FGFR2 activity by IHC in human tissue array, the result revealed that FGFR2 expression was positively correlated with pSTAT3 and pERK1/2 (Figure 6F).

MDA‐MB‐231 cells with FGFR2‐S252W‐stable expression (231‐S252W) treated with the FGFR2 inhibitors AZD4547 and BGJ398 to elucidate the molecular mechanism by which FGFR2 signaling induces PD‐L1 expression. Both inhibitors suppressed PD‐L1 expression, which in turn, downregulated the expression of pSTAT3 and pERK (Figure 6G). Thus, STAT3 and ERK may be implicated in FGFR2‐mediated PD‐L1 regulation. We then treated the 231‐S252W cells with the STAT3 inhibitor (C188‐9) and the ERK inhibitor (U0126) and confirmed that both agents suppressed PD‐L1 expression in a dose‐dependent manner (Figure 6H,I). C188‐9 (at concentrations exceeding 1 × 10^−6^ m) downregulated the expression of pSTAT3, pERK1/2, and PD‐L1. Hence, STAT3 may regulate PD‐L1 via ERK1/2. At a concentration of 1 × 10^−6^ m, U0126 began to inhibit pERK and PD‐L1, but it downregulated pSTAT3 at a concentration of 20 × 10^−6^ m (Figure 6I). Thus, ERK signaling might regulate STAT3 through a feedback mechanism.

These data suggest that STAT3 and ERK are involved in PD‐L1 regulation through FGFR2. To further investigate this, we cotreated cells with STAT3i and ERKi at various concentrations and found that combinations of both inhibitors elicited stronger effects than either inhibitor alone. Furthermore, 1 × 10^−6^ m STAT3i and 5 × 10^−6^ m ERKi significantly suppressed PD‐L1 expression (Figure 6J).

Peng^[^ ^26^ ^]^ reported that high PD‐L1 expression levels limit antitumor immune responses. Considering PD‐L1 upregulation in Fgfr2‐S252W, we examined the immunological changes associated with Fgfr2 activation in *Brca1‐MKO* mice. Flow cytometry with different markers revealed significantly fewer activated CD8^+^ T cells in *Fgfr2‐S252W;Brcal‐MKO* tumors than *Fgfr2‐S252W* or *Brca1‐MKO* tumors (Figure 6K–N; and Figure S5A, Supporting Information). In contrast, IHC (Figure S5A, Supporting Information), RT‐qPCR (Figure S5B,D, Supporting Information), and flow cytometry (Figure 6K–N; and Figure S5F,G, Supporting Information) showed that the *Fgfr2‐S252W;Brca1‐MKO* tumors displayed unbalanced M1:M2 macrophage ratios compared with the *Fgfr2‐S252W* and *Brca1‐MKO* mammary tumors. M2 macrophages help generate an immunosuppressive environment that blocks T cell activation. The immunosuppressive cytokines IFN‐*γ*, TGF‐*β*, and IL10 were significantly upregulated in the *Fgfr2‐S252W;Brcal‐MKO* tumors compared with the *Fgfr2‐S252W* tumors (Figure S5A,C,E, Supporting Information). Thus, upregulation of multisignaling triggered by FGFR2 activation collaborated with Brca1 deficiency creates an immunosuppressive environment that enhances tumor progression.

### 2.7 Inhibition of FGFR2 Signaling Suppresses BRCA1‐Deficient Tumor Progression

As Fgfr2 activation enhances Brca1‐deficient tumor formation, we explored whether Fgfr2 inhibition is a viable therapeutic option for Brca1‐associated tumors. We generated isogenic cancer pairs by using lentiviral vectors encoding two individual short‐hairpin RNAs (shRNA) against Brca1 to transduce a cell line derived from a Fgfr2‐S252W mammary tumor (**Figure** **7**A,B). All cell lines with Fgfr2‐S252W and Fgfr2‐S252W+shBrca1 were equally sensitive to the FGFR inhibitor (FGFRi) BGJ398 under cell culture conditions (Figure 7C). We then established allograft tumors by implanting these cells into the fourth mammary fat pads of nude mice. When the tumors were established, we compared their growth in the animals treated with BGJ398 against those in the untreated mice. In the absence of BGJ398, the Fgfr2‐S252W+shBrca1‐1, and Fgfr2‐S252W+shBrca1‐2 tumors grew more than the Fgfr2‐S252W tumors. However, BGJ398 significantly inhibited all tumor growth (Figure 7D,E). No treatment caused any significant body weight loss during the study period (Figure 7F). The tumors were subjected to anti‐Ki67 and anticleaved caspase‐3 antibodies and presented with markedly decreased proliferation and increased apoptosis in response to BGJ398 (Figure 7G). These data confirmed that FGFR2 signaling promotes and maintains BRCA1‐deficient tumor progression. Moreover, FGFRi suppressed STAT3‐ERK activity as well as Snail and *N*‐cadherin expression but upregulated YY1 (Figure 7H). Therefore, FGFR2 inhibition could profoundly affect tumor growth.

Figure 7Establishment of a tumor slice culture platform for rapid evaluation of the therapeutic efficacy of anticancer drugs. A,B) Fgfr2‐S252W tumor cells were lentiviral transduced with shRNA for depletion of Brca1 (shBr‐1 and shBr‐2) or a scrambled control shRNA. Brca1 levels were assessed by RT‐PCR A) and WB B) immediately after selection. C) Alamar blue assay evaluating the effect of BGJ398 on proliferation of the indicated mouse cell lines. D–F) Growth/weight curves of tumors in mice treated with BGJ398 (*n* = 6). BGJ398 alone results in tumor remission D,E) and weight change F). G) H&E and Immunohistochemical staining of sections showing that the BGJ398 treatment suppressed the expression of Ki67 and induced Cleaved Casp3. H) Representative WB analysis reveals that BGJ398 inhibits FRS2, ERK1/2, STAT3 phosphorylation and downstream signaling pathway activation. I,J) Visual antitumor response and improved immunotherapy with the combination of FGFR inhibitor and anti‐PD‐1/PD‐L1 in tumor slice culture system revealed by MTT (3‐(4,5‐dimethylthiazol‐2‐yl)‐2,5‐diphenyltetrazolium bromide) analysis. K,L) MTT analysis of tumor tissue slices prepared from *Fgfr2‐S252W* spontaneous models to show their responses to various cytotoxic and targeted therapeutics. M) IHC staining against IFN*γ*, CD8, Cleaved Casp3, Ki67, and PD‐L1 with indicated treatment groups. Data represent the mean ± SEM . *p* values were determined by ANOVA with Tukey's multiple comparison test A, E, K, and L). Statistical analysis was carried out using GraphPad Prism 7 Software. *p < 0.05, **p < 0.01, ***p < 0.001, ****p < 0.0001.

**Figure:**
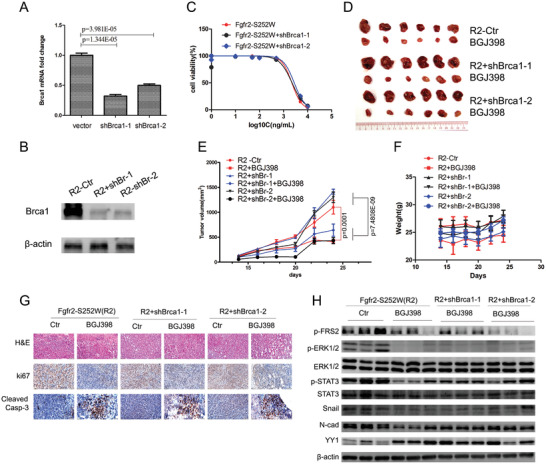

**Figure:**
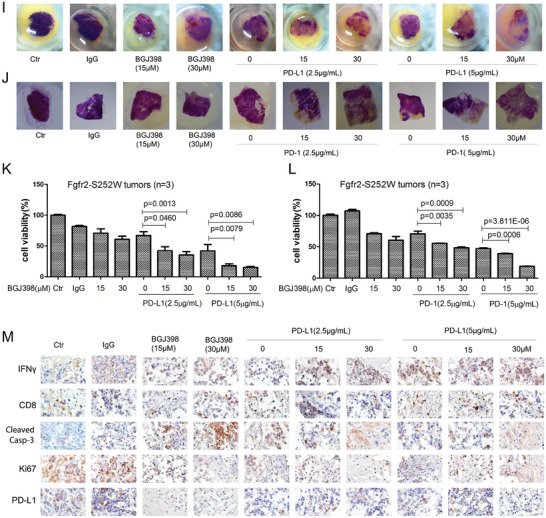

### 2.8 Establishment of a Tumor Slice Culture Platform for Rapid Evaluation of the Therapeutic Efficacy of Anticancer Drugs

It is essential to develop quick, reliable models to validate the clinical and therapeutic efficacy of individual and combinations of anticancer drugs. *Fgfr2‐S252W* and *Fgfr2‐S252W;Brca1^–1–^* tumors are sensitive to FGFRi but grow only very slowly. We used them to establish a platform for rapid assessment of the therapeutic efficacy of anticancer drugs. We prepared slices of mammary tumors excised from *Fgfr2‐S252W* mice (Figure S6A, Supporting Information). According to the MTT (3‐(4,5‐dimethylthiazol‐2‐yl)‐2,5‐diphenyltetrazolium bromide) assay (Figure S6B,C, Supporting Information), slice viability was about 90% compared with the primary tumors in the first 5 days and declined to about 80% after 5–7 days. We treated the tumor slices with various concentrations of BGJ398 for 4 days and compared their tissue viability. BGJ398 treatment increased lethality in a dose‐dependent manner (Figure S6D,E, Supporting Information). We also prepared slices of mammary tumors developed in a transgenic mouse strain bearing *MMTV‐cNeu* genes that activate PI3K.^[^ ^27^, ^28^ ^]^ We subjected the tumor slices to the PI3K inhibitor BKM120 and the FGFRi inhibitors AZD4547 and BGJ398. FGFRi had negligible effect on the *MMTV‐cNeu* mammary tumors. However, BKM120 killed the tumor cells in a dose‐dependent manner (Figure S6F, Supporting Information). Therefore, tumor slice culture is a good platform for targeted therapy screening.

Immune checkpoint blockade (ICB) targets PD‐1 and/or PD‐L1 and is promising as a cancer therapy. Nevertheless, it only benefits about 20% of all patients.^[^ ^29^ ^]^ Our tumor slice platform rapidly assesses anticancer drug efficacy. We examined whether it could be used to evaluate the efficiency of ICB mediated by anti‐PD‐1 and anti‐PD‐L1 antibodies. Both anti‐PD‐1 and anti‐PD‐L1 killed cancer cells in a dose‐dependent manner at 4 days after treatment (Figure S6G–J, Supporting Information). We then investigated the impact of FGFR2i on ICB and found that the administration of 15 and 30 × 10^−6^ m FGFR2i significantly enhanced the killing efficacy of 2.5 and 5 *μ*g *α*PD‐L1 revealed by MTT (3‐(4,5‐dimethylthiazol‐2‐yl)‐2,5‐diphenyltetrazolium bromide) analysis (Figure 7I,K). The combination of 5 *μ*g *α*PD‐1 and FGFRi had far greater efficacy than the combination of 2.5 *μ*g *α*PD‐1 and FGFRi (Figure 7J,L).

We measured the IFN*γ*, CD8^+^, cleaved caspase 3, Ki67, and PD‐L1 protein levels by IHC staining to uncover the mechanism underlying tumor growth suppression by FGFR inhibitor and immunotherapy (Figure S6K, Supporting Information). Figure 7M shows that the CD8, Ki67, and PD‐L1 levels did not diminish after 4 days culture. In contrast, the PD‐L1 and Ki67 levels dramatically decreased and the INF‐*γ* and CD8 levels markedly increased in the anti‐PD‐L1 group compared with the control. Hence, the tumor slice culture system can rapidly assess the efficacy of the combination of FGFR inhibitor and PD‐1/PD‐L1 blockade in patients with FGF/FGFR‐driven tumors.

## 3 Discussion

A genome‐wide association study (GWAS) revealed that FGFR2, TNRC9, MAP3K1, and LSP1 were strongly associated with breast cancer risk.^[^ ^30^ ^]^ A GWAS of invasive breast cancer in postmenopausal white women associated several *FGFR2* alleles with the risk of sporadic postmenopausal breast cancer.^[^ ^4^ ^]^ Other studies disclosed that FGFR2 is frequently upregulated in human breast cancers.^[^ ^6^, ^31^ ^]^ Despite evidence implicating FGFR2 in breast cancer development, it is unknown whether FGFR2 activation induces breast cancer. In the present study, we generated the conditionally activated point mutation Fgfr2‐S252W that has also been observed in human breast cancer.^[^ ^32^, ^33^ ^]^ We investigated the possible role of Fgfr2 activation in mammary tumor formation. Our data indicated that FGFR2 activation in mammary tissues induced tumor formation in 30.13% (22/73) of all mice and the median time was 22 months. The mutant strain revealed that 1) FGFR2 activation significantly increases the incidence of TNBC formation, 2) tumorigenesis is accompanied by BRCA1 downregulation and is accelerated by targeted BRCA1 deletion, 3) FGFR2 activation upregulates PD‐L1 associated with pSTAT3, and 4) FGFR2 inhibition and anti‐PD‐1/anti‐PD‐L1 blockade markedly inhibit tumor growth. These observations provide strong evidence for the involvement of FGFR2 activation in breast cancer formation and demonstrate a novel putative therapeutic option for breast cancer associated with FGF/FGFR2 activation.

TNBC occurs in about 15–20% of all human breast cancer cases.^[^ ^34^, ^35^, ^36^ ^]^ TNBCs are refractory to currently available therapies. Hence, it is vital to learn their origins and the main factors driving their formation. Changes in the expression levels of the tumor suppressors and oncogenes BARD1, BRCA1, BRCA2, FGFR2, p53, PALB2, RAD51D, and PI3K as well as many other genes are correlated with TNBC.^[^ ^35^, ^37^, ^38^ ^]^ Nevertheless, the gene lists reported in these studies seldom overlapped. Hence, most of these genes might only be markers for the TNBCs characteristic of various populations. In the present study, we found that 56.25% (18/32) of all the mammary tumors that developed in the Fgfr2‐S252W mice were TNBCs. Thus, activated FGF/FGFR2 signaling could promote TNBC formation. We also analyzed mammary tumors developed in Brca1 mutant mice and discovered that 45.83% (11/24) of them were TNBCs. Characterization of the TNBCs developed in these mouse models and identification of their similarities and differences might help evaluate them for future translational work.

One of the notable findings in this study is that Fgfr2 suppresses expression of BRCA1, which is a well‐known breast cancer suppressor gene in humans and mice.^[^ ^25^, ^39^, ^40^ ^]^ About one‐third of all sporadic breast cancers have low BRCA1 levels even though they are WT for BRCA1.^[^ ^41^ ^]^ Thus, BRCA1 may undergo transcriptional regulation. We previously showed that BRCA1 expression is suppressed by the transcriptional factor YY1.^[^ ^23^ ^]^ Here, we found that FGFR2 activation suppresses YY1 expression which, in turn, accounts for reduced BRCA1 expression. This phenomenon might explain the similar rates of TNBC development in both Fgfr2‐S252W and Brca1 mutant mice. To confirm that FGFR2 signaling triggers mammary tumorigenesis is partially mediated by Brca1 suppression, we disrupted Brca1 and found that *Fgfr2‐S252W*;*Brca1‐MKO* double mutant mice exhibited faster tumorigenesis and slightly higher TNBC frequency (62.50%) than mice bearing the single mutations.

It was recently shown that FGFR2 promotes PD‐L1 expression via the JAK/STAT3 signaling pathway in colorectal cancer.^[^ ^42^ ^]^ Our data revealed that Fgfr2‐S252W expression in mammary tissues upregulated PD‐L1. We also showed that ERK and STAT3 coinhibition more effectively suppressed PD‐L1 expression than either inhibition factor alone. Thus, the regulation of PD‐L1 expression by Fgfr2 may be jointly mediated by pSTAT3 and ERK signaling.

Earlier research showed that targeting the immune checkpoint mediated by PD‐1/PD‐L1 reactivated preexisting antitumor immune responses and demonstrated clinical efficacy. However, it was only beneficial for a subset of the patients.^[^ ^43^ ^]^ Thus, it is vital to identify combinations of anticancer drugs that can enhance ICB. In March 2019, the Food and Drug Administration (FDA) of the United States granted accelerated approval for the administration of the atezolizumab‐paclitaxel combination to adult patients with unresectable locally advanced or metastatic TNBC expressing PD‐L1. Here, we found that the combination of FGFR2 signaling inhibition with BGJ398^[^ ^44^ ^]^ with anti‐PD‐L1 or anti‐PD‐1 antibodies was highly efficacious. Moreover, our tumor slice culture platform disclosed that drug sensitivity tests could be completed in 4 days. This system is potentially useful for validating the sensitivity of human breast cancers to combinatory anticancer drug and ICB therapy.

The present study provides empirical evidence that FGFR2 and the FGF signaling mediated by it are implicated in mammary tumorigenesis. Our analysis revealed that FGFR2 activation induces the ERK and STAT3 pathways, upregulates PD‐L1, and promotes EMT. FGFR2 activation also downregulates BRCA1 via the FRS2*α*/STAT3/MAPK pathways mediated by the transcription factor YY1. The foregoing responses are jointly responsible for mammary tumorigenesis. We also used a tumor slice platform to demonstrate that a combination of FGFR inhibitor plus ICB mediated by PD‐1 and PD‐L1 effectively inhibits FGFR2‐induced tumorigenesis. As FGFR2 is widely expressed in human breast cancers, this discovery is a putative novel therapeutic option for human tumors expressing FGFR2 and PD‐1 or PD‐L1.

## 4 Experimental Section

### Animals

Mice carrying mammary specific activation of Fgfr2 (Fgfr2‐S252W) were generated by breeding the previously generated *Fgfr2^pLoxpneo‐S252W/+^* mice^[^ ^14^ ^]^ and *MMTV‐Cre* mice.^[^ ^18^ ^]^ The Fgfr2‐S252W mice were then crossed with the *Brca1^co/co^;MMTV‐Cre* (Brca1‐MKO) mice^[^ ^25^ ^]^ to generate Fgfr2/Brca1 double mutant mice (Fgfr2‐S252W;Brca1‐MKO). The Fgfr2‐S252W mice, Brca1‐MKO mice, and Fgfr2‐S252W;Brca1‐MKO mice were in mixed genetic background of 129SVEV:Black Swiss:FVB at roughly 1:1:2 ratio. All animal experiments were approved by the University of Macau Animal Ethics Committee under Protocol No. UMAEC‐037‐2015).

### Cell Culture and Viral Infection

The 293T, MDA‐MB‐231, MCF7, MDA‐MB‐436, MDA‐MB‐468, and T‐47D cell lines were obtained from the American Type Culture Collection (ATCC, Manassas, VA). All cells except *Fgfr2‐S252W* mammary gland and mammary tumor lines were grown in F‐medium.^[^ ^45^ ^]^ CRISPR‐Cas9 lentivector infections were performed using a previously described general protocol. Unique sgRNA sequences were individually cloned into the lenti‐CRISPR v2 vector (Addgene plasmid No. 52 961) containing a puromycin resistance marker. The sgRNA sequences for each vector are listed in Table S1 (Supporting Information). For lentivirus production, 293T cells (7 × 10^6^) were seeded in 10 cm plates at 12 h before transfection in Iscove modified Dulbecco's culture medium with 10% v/v fetal bovine serum, 100 IU mL^−1^ penicillin, and 100 mg mL^−1^ streptomycin in a 5% CO_2_, 370C incubator. 9 *μ*g plasmid DNA was used per dish and consisted of pMD2‐VSVG envelope plasmid (3 µg) (Addgene plasmid No. 8454), pCMVR8.2 packaging plasmid (2 µg) (Addgene plasmid No. 12 263), and CRISPR‐Cas9 lentiCRISPRV2 plasmid DNA (4 µg). After 48 h, the lentivirus‐containing supernatants were passed through a 0.45 µm filter and concentrated with Lenti‐X Concentrator (PT4421‐2; Clontech Laboratories, Mountain View, CA) following the manufacturer's protocol.

A portion of the freshly prepared lentivirus was used to determine the viral titer. Briefly, FGFR2 mammary gland tumor cells were infected for 2 days with various volumes of viral stock and then subjected to puromycin (Invitrogen, Carlsbad, CA) selection (2 µg mL^−1^) for 2 days. Cell survival was measured and recorded as the infection rate. Based on the titer, an adjusted amount of virus stock was used to infect FGFR2 mammary gland tumor cells at a target MOI = 0.2. In this manner, most cells incorporated fewer than two lentivirus particles. 2 days after infection, puromycin (Invitrogen, Carlsbad, CA) was added at a final 4 µg mL^−1^ concentration to select infected cells for 2 days. The selected cells were then transferred to F‐medium for the subsequent assay.

### Plasmids

The pBp‐FGFR2b‐WT (Addgene plasmid No. 45 698) and pBp‐FGFR2c‐WT (Addgene plasmid No. 45 699) were gifts from Matthew Meyerson. The BRCA1 luciferase reporters were requested from Dr. Mi‐Hye Lee and the FGFR2‐S252W plasmid was constructed in the CD511B‐1 vector. The other plasmids used were pcDNA3.1 HA‐YY1 (Addgene plasmid No. 104 395), lentiCRISPR v2 (Addgene plasmid No. 52 961), pCMV‐VSV‐G (Addgene plasmid No. 8454), pCMV‐dR8.2 dvpr (Addgene plasmid No. 8455), pLKO‐shBRCA1 #1 (Addgene plasmid No. 44 594), and pLKO‐shBRCA1 #2 (Addgene plasmid No. 44 595).

### Transfection and Luciferase Assay

Transfections were performed using Lipofectamine 3000 (L3000015; Thermo Fisher Scientific, Waltham, MA) according to the manufacturer's instructions. Cells were harvested 24 h post‐transfection and luciferase activity was assayed with a dual luciferase reporter assay system (Promega, Madison, WI). Renilla luciferase activity was used for normalization.

### Chromatin Immunoprecipitation (ChIP) and BRCA1 Promoter Analysis

ChIP assays were performed as previously described.^[^ ^23^, ^46^ ^]^ Cells were cross‐linked with 1% v/v formalin. DNA was extracted from YY1 immunoprecipitates. For the PCR, a 2 µL aliquot of DNA extract (30 µL) and VENT polymerases (Biolabs, Northbrook, IL) were used. Anti‐YY1 antibody was purchased from Santa Cruz Biotechnology (Dallas, TX). The ChIP primers for the BRCA1 promoter are listed in Table S1 (Supporting Information).

### Quantitative Real‐Time (qRT)‐PCR and Western Blot Analysis

Total RNA was isolated from tumors/mammary gland/cells with TRIzol reagent (Thermo Fisher Scientific, Waltham, MA) according to the manufacturer's instructions. Reverse transcription was performed with a QuantiTect reverse transcription kit (205 313; Qiagen, Hilden, Germany). RT‐PCR was performed with FastStart Universal SYBR Green Master (4 913 850 001; Roche Diagnostics, Basel, Switzerland) in a QuantStudio 7 Flex real‐time PCR system (Thermo Fisher Scientific, Waltham, MA). Relative quantitation was achieved by normalization to 18S. The primers used for RT‐PCR are listed in Table S1 (Supporting Information). Tumors/mammary glands/cells were washed in phosphate‐buffered saline and lysed in radioimmunoprecipitation assay (RIPA) buffer (10 × 10^−3^ m Tris‐HCl (pH 8.0), NaCl (150 × 10^−3^ m), Triton X‐100 (1%), deoxycholic acid (1%), SDS (0.1%), and protease inhibitor cocktail) as previously described. Western blot was conducted in a ChemiDoc MP imaging system (Bio‐Rad Laboratories, Hercules, CA) using the corresponding antibodies. Band intensity was quantified with ImageJ (National Institutes of Health (NIH), Bethesda, MD). The numbers under the immunoblots represented the intensity relative to the first band. Relative quantitation was achieved by normalization to *β*‐actin. The antibodies used in the Western blot are listed in Table S2 (Supporting Information).

### Whole Mounts, H&E, IHC, and IF Staining

The fourth abdominal mammary glands were harvested from pubertal mice, placed between two glass slides, spread out by placing weights on top of the slides, and fixed in EtOH:CHCl3:HOAC (6:3:1, v/v) as previously described.^[^ ^25^ ^]^ The tumors/mammary glands were fixed with paraformaldehyde (4% v/v) and stained with corresponding antibodies according to previously described methods. Deparaffinized thin sections of the tumors/mammary glands were cooked with Retriever (62700‐10; Electronic Microscopy Sciences, Hatfield, PA) in Buffer A (citrate; pH 6.0) followed by antibody staining. Images were acquired with a Nikon A1R confocal system (Nikon Corp., Tokyo, Japan) or an Olympus IX83 inverted microscope (Olympus Corp., Tokyo, Japan). Fluorescent signal intensity, area, and colocalization were analyzed with ImageJ (National Institutes of Health (NIH), Bethesda, MD). The antibodies used in IHC/IF staining are listed in Table S2 (Supporting Information).

### Tumor Digestion and Flow Cytometry

Tumors and mammary glands were isolated, minced, and digested with Digestion I at 37 °C for 3–4 h. The cells were spun down and treated with Digestion II for 5 min. Digested tumor and mammary gland cells were washed with Hanks’ solution and lysed with red blood cell (RBC) lysis solution. The cells were resuspended in flow cytometry staining buffer (Thermo Fisher Scientific, Waltham, MA) and incubated with antibodies (Table S3, Supporting Information) on ice for 1 h. The cells were then washed and resuspended in flow cytometry staining buffer and analyzed by flow cytometry (FACSAria III; BD BioScience, Franklin Lakes, NJ). The signal threshold was defined using all‐stained, unstained, and isotype controls.

### RNA‐Seq and Analysis and Gene Set Enrichment Analysis (GSEA)

Twenty‐four RNA‐seq libraries from mammary gland tumors (*Fgfr2‐WT* (three samples), *Fgfr2‐S252W* (three samples), and *Fgfr2‐S252W* (18 samples)) were constructed with an Illumina TruSeq RNA library prep kit (Illumina, San Diego, CA) and sequenced on an Illumina HiSeq X‐Ten platform (Illumina, San Diego, CA) to generate over 25 million paired‐end, 150‐bp reads. Sequencing reads were aligned to the mouse reference genome mm10 and processed with HISAT2 v. 2.1.0 ^[^ ^47^ ^]^ Differential expression analysis was conducted with DESeq2 v. 1.22.2 ^[^ ^48^ ^]^ Reads from each target gene exon were counted and then normalized with the total reads and lengths of the individual exons to avoid bias caused by variations in exon size. GSEA was performed using the R package clusterProfiler.^[^ ^49^ ^]^ Briefly, the enrichment score (ES) was calculated as the maximum deviation from zero of the weighted fraction of genes present minus the fraction not present up to a given index in a gene expression matrix ordered by phenotype correlation. The statistical significance (nominal *p* value) of the ES of a gene set was estimated using an empirical gene‐based permutation test. The normalized enrichment score was calculated by creating 1000 permutations of the ES and scaling the observed ES by the mean score of the permutations.

### Organotypic Slice Culture

A tumor slice culture system^[^ ^50^ ^]^ was applied in this experiment. Three solutions were required to prepare the reconstituted collagen gel. Solution A was type 1 collagen (Cultrex 3D culture matrix rat collagen I; R&D Systems, Minneapolis, MN) and it was stored at 4 °C. Solution B was 10× concentrated sterile culture medium (Ham's F‐12 nutrient mix powder; Gibco; Grand Island, NY). The Ham's F‐12 powder was dissolved in sterile Milli‐Q water, passed through a 0.22 µm Corning filter (Corning, NY), and stored at 4 °C. Solution C was sterile reconstitution buffer. To prepare 100 mL solution, sterile Milli‐Q water (100 mL), NaOH (0.05 m), HEPES (200 × 10^−3^ m), and NaHCO_3_ (2.2 g) were mixed. Solutions A, B, and C were mixed at 8:1:1 v/v/v ratio to make reconstituted collagen gel. The culture medium consisted of F‐medium (500 mL), Dulbecco's modified Eagle's medium (DMEM; 373 mL), insulin (5 µg mL^−1^), amphotericin B (250 ng mL^−1^), gentamicin (10 µg mL^−1^), cholera toxin (0.1 × 10^−9^ m), EGF (0.125 ng mL^−1^), hydrocortisone (25 ng mL^−1^), and ROCK inhibitor (10 × 10^−6^ m). BGJ398 and AZD4547 (FGFR inhibitors) were stored in dimethyl sulfoxide (DMSO) at −20 °C before use. They were then diluted with culture medium to 1, 15, and 30 × 10^−6^ m. Anti‐PDL1 and anti‐PD1 were diluted with culture medium to 1.25, 2.5, and 5 × 10^−6^ m. Immunoglobulin G (IgG) was diluted to 5 × 10^−6^ m.

### Statistical Analysis

All values are means ± SEM of individual samples. The samples were analyzed by unpaired two‐tailed *t*‐tests or by one/two‐way ANOVA. Sample size (*n*) for each statistical analysis was given in the corresponding text. *p* < 0.05 was considered statistically significant. Correlations were identified by Pearson's correlation test. All analysis were conducted in GraphPad Prism 7 (GraphPad Software, La Jolla, CA).

## Conflict of Interest

The authors declare no conflict of interest.

## Author Contributions

C.X.D. designed and provided guidance for this study. J.H.L., M.H.L., and Z.H acquired the data. C.X.D. and J.H.L. wrote the manuscript. J.H.L. M.H.L., K.M., Z.H., Z.Y., X.J., M.Z., X.Z., S.C., NG.J., Y.F., F.X., P.C., and H.S. conducted the experiments. A.Z., analyzed the RNA‐sequencing data and performed other bioinformatics analyses. P.C. provided the tumor organoids. Z.Y. and Z.H. provided human tissue array. C.X.D., X.X., L.C., T.X., and Q.C. supervised the experiments.

## Supporting information

Supporting Information

## Data Availability

The data that supports the findings of this study are available in the supplementary material of this article.

## References

[1] A. Jemal , F. Bray , M. M. Center , J. Ferlay , E. Ward , D. Forman , Ca‐ Cancer J. Clin. 2011, 61, 69. 21296855 10.3322/caac.20107

[2] I. Godet , D. M. Gilkes , Integr. Cancer Sci. Ther. 2017, 4, 10.15761. 10.15761/ICST.1000228 PMC5505673 28706734

[3] D. F. Easton , K. A. Pooley , A. M. Dunning , P. D. P. Pharoah , D. Thompson , D. G. Ballinger , J. P. Struewing , J. Morrison , H. Field , R. Luben , N. Wareham , S. Ahmed , C. S. Healey , R. Bowman , K. B. Meyer , C. A. Haiman , L. K. Kolonel , B. E. Henderson , L. Le Marchand , P. Brennan , S. Sangrajrang , V. Gaborieau , F. Odefrey , C. Y. Shen , P. E. Wu , H. C. Wang , D. Eccles , D. G. Evans , J. Peto , O. Fletcher , N. Johnson , S. Seal , M. R. Stratton , N. Rahman , G. Chenevix‐Trench , S. E. Bojesen , B. G. Nordestgaard , C. K. Axelsson , M. Garcia‐Closas , L. Brinton , S. Chanock , J. Lissowska , B. Peplonska , H. Nevanlinna , R. Fagerholm , H. Eerola , D. Kang , K. Y. Yoo , D. Y. Noh , S. H. Ahn , D. J. Hunter , S. E. Hankinson , D. G. Cox , P. Hall , S. Wedren , J. J. Liu , Y. L. Low , N. Bogdanova , P. Schurmann , T. Dork , R. A. E. M. Tollenaar , C. E. Jacobi , P. Devilee , J. G. M. Klijn , A. J. Sigurdson , M. M. Doody , B. H. Alexander , J. H. Zhang , A. Cox , I. W. Brock , G. MacPherson , M. W. R. Reed , F. J. Couch , E. L. Goode , J. E. Olson , H. Meijers‐Heijboer , A. van den Ouweland , A. Uitterlinden , F. Rivadeneira , R. L. Milne , G. Ribas , A. Gonzalez‐Neira , J. Benitez , J. L. Hopper , M. McCredie , M. Southey , G. G. Giles , C. Schroen , C. Justenhoven , H. Brauch , U. Hamann , Y. D. Ko , A. B. Spurdle , J. Beesley , X. Q. Chen , A. Mannermaa , V. M. Kosma , V. Kataja , J. Hartikainen , N. E. Day , D. R. Cox , B. A. J. Ponder , S. Collaborators , kConFab , A. M. Grp , Nature 2007, 447, 1087.17529967

[4] D. J. Hunter , P. Kraft , K. B. Jacobs , D. G. Cox , M. Yeager , S. E. Hankinson , S. Wacholder , Z. Wang , R. Welch , A. Hutchinson , J. Wang , K. Yu , N. Chatterjee , N. Orr , W. C. Willett , G. A. Colditz , R. G. Ziegler , C. D. Berg , S. S. Buys , C. A. McCarty , H. S. Feigelson , E. E. Calle , M. J. Thun , R. B. Hayes , M. Tucker , D. S. Gerhard , J. F. Fraumeni Jr. , R. N. Hoover , G. Thomas , S. J. Chanock , Nat. Genet. 2007, 39, 870. 17529973 10.1038/ng2075 PMC3493132

[5] H. Liu , C. J. Murphy , F. A. Karreth , K. B. Emdal , F. M. White , O. Elemento , A. Toker , G. M. Wulf , L. C. Cantley , Cancer Discovery 2018, 8, 354. 29203461 10.1158/2159-8290.CD-17-0679 PMC5907916

[6] H. Lei , C. X. Deng , Int. J. Biol. Sci. 2017, 13, 1163. 29104507 10.7150/ijbs.20792 PMC5666331

[7] N. Turner , R. Grose , Nat. Rev. Cancer 2010, 10, 116. 20094046 10.1038/nrc2780

[8] N. Turner , M. B. Lambros , H. M. Horlings , A. Pearson , R. Sharpe , R. Natrajan , F. C. Geyer , M. van Kouwenhove , B. Kreike , A. Mackay , A. Ashworth , M. J. van de Vijver , J. S. Reis‐Filho , Oncogenes 2010, 29, 2013. 10.1038/onc.2009.489 PMC2852518 20101236

[9] C. Sun , O. I. Olopade , A. Di Rienzo , Cancer Genet. Cytogenet. 2010, 197, 193. 20193855 10.1016/j.cancergencyto.2009.11.006 PMC2831800

[10] M. Katoh , Trends Pharmacol. Sci. 2016, 37, 1081. 27992319 10.1016/j.tips.2016.10.003

[11] F. Guffanti , R. Chila , E. Bello , M. Zucchetti , M. Zangarini , L. Ceriani , M. Ferrari , M. Lupi , A. Jacquet‐Bescond , M. F. Burbridge , M. J. Pierrat , G. Damia , Neoplasia 2017, 19, 35. 27988457 10.1016/j.neo.2016.11.008 PMC5167242

[12] S. Sun , Y. Jiang , G. Zhang , H. Song , X. Zhang , Y. Zhang , X. Liang , Q. Sun , D. Pang , J. Surg. Oncol. 2012, 105, 773. 22006548 10.1002/jso.22120

[13] H. J. Lee , A. N. Seo , S. Y. Park , J. Y. Kim , J. Y. Park , J. H. Yu , J. H. Ahn , G. Gong , Ann. Surg. Oncol. 2014, 21, 1561. 24385208 10.1245/s10434-013-3456-x

[14] L. Chen , D. Li , C. L. Li , A. Engel , C. X. Deng , Bone 2003, 33, 169. 14499350 10.1016/s8756-3282(03)00222-9

[15] J. Anderson , H. D. Burns , P. Enriquez‐Harris , A. O. Wilkie , J. K. Heath , Hum. Mol. Genet. 1998, 7, 1475. 9700203 10.1093/hmg/7.9.1475

[16] O. A. Ibrahimi , F. Zhang , A. V. Eliseenkova , N. Itoh , R. J. Linhardt , M. Mohammadi , Hum. Mol. Genet. 2004, 13, 2313. 15282208 10.1093/hmg/ddh235 PMC4140565

[17] V. Shukla , X. Coumoul , R. H. Wang , H. S. Kim , C. X. Deng , Nat. Genet. 2007, 39, 1145. 17694057 10.1038/ng2096

[18] K. U. Wagner , R. J. Wall , L. St‐Onge , P. Gruss , A. Wynshaw‐Boris , L. Garrett , M. Li , P. A. Furth , L. Hennighausen , Nucl. Acids Res. 1997, 25, 4323. 9336464 10.1093/nar/25.21.4323 PMC147032

[19] A. Vassilopoulos , R. H. Wang , C. Petrovas , D. Ambrozak , R. Koup , C. X. Deng , Int. J. Biol. Sci. 2008, 4, 133. 18461147 10.7150/ijbs.4.133 PMC2367429

[20] Y. Zhou , B. Zhou , L. Pache , M. Chang , A. H. Khodabakhshi , O. Tanaseichuk , C. Benner , S. K. Chanda , Nat. Commun. 2019, 10, 1523. 30944313 10.1038/s41467-019-09234-6 PMC6447622

[21] D. Glodzik , A. Bosch , J. Hartman , M. Aine , J. Vallon‐Christersson , C. Reutersward , A. Karlsson , S. Mitra , E. Nimeus , K. Holm , J. Hakkinen , C. Hegardt , L. H. Saal , C. Larsson , M. Malmberg , L. Ryden , A. Ehinger , N. Loman , A. Kvist , H. Ehrencrona , S. Nik‐Zainal , A. Borg , J. Staaf , Nat. Commun. 2020, 11, 3747. 32719340 10.1038/s41467-020-17537-2 PMC7385112

[22] K. Miao , J. H. Lei , M. V. Valecha , A. Zhang , J. Xu , L. Wang , X. Lyu , S. Chen , Z. Miao , X. Zhang , S. M. Su , F. Shao , B. K. Rajendran , J. Bao , J. Zeng , H. Sun , P. Chen , K. Tan , Q. Chen , K. H. Wong , X. Xu , C. X. Deng , Nat. Commun. 2020, 11, 3256. 32591500 10.1038/s41467-020-16936-9 PMC7320176

[23] M. H. Lee , T. Lahusen , R. H. Wang , C. Xiao , X. Xu , Y. S. Hwang , W. W. He , Y. Shi , C. X. Deng , Oncogene 2012, 31, 116. 21666725 10.1038/onc.2011.217 PMC9421919

[24] F. Shao , X. Lyu , K. Miao , L. Xie , H. Wang , H. Xiao , J. Li , Q. Chen , R. Ding , P. Chen , F. Xing , X. Zhang , G. H. Luo , W. Zhu , G. Cheng , N. W. Lon , S. E. Martin , G. Wang , G. Chen , Y. Dai , C. X. Deng , Adv. Sci. 2020, 7, 2001914. 10.1002/advs.202001914 PMC7709997 33304752

[25] X. L. Xu , K. U. Wagner , D. Larson , Z. Weaver , C. L. Li , T. Ried , L. Hennighausen , A. Wynshaw‐Boris , C. X. Deng , Nat. Genet. 1999, 22, 37. 10319859 10.1038/8743

[26] Q. Peng , X. Qiu , Z. Zhang , S. Zhang , Y. Zhang , Y. Liang , J. Guo , H. Peng , M. Chen , Y. X. Fu , H. Tang , Nat. Commun. 2020, 11, 4835. 32973173 10.1038/s41467-020-18570-x PMC7518441

[27] C. T. Guy , R. D. Cardiff , W. J. Muller , J. Biol. Chem. 1996, 271, 7673. 8631805 10.1074/jbc.271.13.7673

[28] E. A. Fry , P. Taneja , K. Inoue , Int. J. Cancer 2017, 140, 495. 27553713 10.1002/ijc.30399 PMC5159240

[29] M. Nishino , N. H. Ramaiya , H. Hatabu , F. S. Hodi , Nat. Rev. Clin. Oncol. 2017, 14, 655. 28653677 10.1038/nrclinonc.2017.88 PMC5650537

[30] D. F. Easton , K. A. Pooley , A. M. Dunning , P. D. Pharoah , D. Thompson , D. G. Ballinger , J. P. Struewing , J. Morrison , H. Field , R. Luben , N. Wareham , S. Ahmed , C. S. Healey , R. Bowman , S. collaborators , K. B. Meyer , C. A. Haiman , L. K. Kolonel , B. E. Henderson , L. Le Marchand , P. Brennan , S. Sangrajrang , V. Gaborieau , F. Odefrey , C. Y. Shen , P. E. Wu , H. C. Wang , D. Eccles , D. G. Evans , J. Peto , O. Fletcher , N. Johnson , S. Seal , M. R. Stratton , N. Rahman , G. Chenevix‐Trench , S. E. Bojesen , B. G. Nordestgaard , C. K. Axelsson , M. Garcia‐Closas , L. Brinton , S. Chanock , J. Lissowska , B. Peplonska , H. Nevanlinna , R. Fagerholm , H. Eerola , D. Kang , K. Y. Yoo , D. Y. Noh , S. H. Ahn , D. J. Hunter , S. E. Hankinson , D. G. Cox , P. Hall , S. Wedren , J. Liu , Y. L. Low , N. Bogdanova , P. Schurmann , T. Dork , R. A. Tollenaar , C. E. Jacobi , P. Devilee , J. G. Klijn , A. J. Sigurdson , M. M. Doody , B. H. Alexander , J. Zhang , A. Cox , I. W. Brock , G. MacPherson , M. W. Reed , F. J. Couch , E. L. Goode , J. E. Olson , H. Meijers‐Heijboer , A. van den Ouweland , A. Uitterlinden , F. Rivadeneira , R. L. Milne , G. Ribas , A. Gonzalez‐Neira , J. Benitez , J. L. Hopper , M. McCredie , M. Southey , G. G. Giles , C. Schroen , C. Justenhoven , H. Brauch , U. Hamann , Y. D. Ko , A. B. Spurdle , J. Beesley , X. Chen , kConFab, A. M. G. , A. Mannermaa , V. M. Kosma , V. Kataja , J. Hartikainen , N. E. Day , D. R. Cox , B. A. Ponder , Nature 2007, 447, 1087.17529967

[31] L. Wein , P. Savas , C. Van Geelen , F. Caramia , K. Moodie , S. Joshi , S. Loi , Ann. Oncol. 2017, 28, 2025. 28430863 10.1093/annonc/mdx194

[32] T. Helsten , S. Elkin , E. Arthur , B. N. Tomson , J. Carter , R. Kurzrock , Clin. Cancer Res. 2016, 22, 259. 26373574 10.1158/1078-0432.CCR-14-3212

[33] P. Priestley , J. Baber , M. P. Lolkema , N. Steeghs , E. de Bruijn , C. Shale , K. Duyvesteyn , S. Haidari , A. van Hoeck , W. Onstenk , P. Roepman , M. Voda , H. J. Bloemendal , V. C. G. Tjan‐Heijnen , C. M. L. van Herpen , M. Labots , P. O. Witteveen , E. F. Smit , S. Sleijfer , E. E. Voest , E. Cuppen , Nature 2019, 575, 210. 31645765 10.1038/s41586-019-1689-y PMC6872491

[34] V. N. R. Gajulapalli , V. L. Malisetty , S. K. Chitta , B. Manavathi , Biosci. Rep. 2016, 36, e00432. 27884978 10.1042/BSR20160228 PMC5180249

[35] Z. Sporikova , V. Koudelakova , R. Trojanec , M. Hajduch , Clin. Breast Cancer 2018, 18, e841. 30146351 10.1016/j.clbc.2018.07.023

[36] C. Shao , M. Yin , L. Deng , P. J. Stambrook , T. Doetschman , J. A. Tischfield , Oncogene 2002, 21, 2840. 11973643 10.1038/sj.onc.1205358

[37] M. D. Burstein , A. Tsimelzon , G. M. Poage , K. R. Covington , A. Contreras , S. A. Fuqua , M. I. Savage , C. K. Osborne , S. G. Hilsenbeck , J. C. Chang , G. B. Mills , C. C. Lau , P. H. Brown , Clin. Cancer Res. 2015, 21, 1688. 25208879 10.1158/1078-0432.CCR-14-0432 PMC4362882

[38] B. D. Lehmann , J. A. Bauer , X. Chen , M. E. Sanders , A. B. Chakravarthy , Y. Shyr , J. A. Pietenpol , J. Clin. Invest. 2011, 121, 2750. 21633166 10.1172/JCI45014 PMC3127435

[39] S. G. Brodie , C. X. Deng , Trends Genet. 2001, 17, S18. 11585672 10.1016/s0168-9525(01)02451-9

[40] C. X. Deng , Environ. Mol. Mutagen. 2002, 39, 171. 11921186 10.1002/em.10069

[41] N. Turner , A. Tutt , A. Ashworth , Nat. Rev. Cancer 2004, 4, 814. 15510162 10.1038/nrc1457

[42] P. Li , T. Huang , Q. Zou , D. Liu , Y. Wang , X. Tan , Y. Wei , H. Qiu , J. Immunol. 2019, 202, 3065. 30979816 10.4049/jimmunol.1801199

[43] P. Sharma , J. P. Allison , Nat. Rev. Immunol. 2020, 20, 75. 31925406 10.1038/s41577-020-0275-8

[44] M. Javle , M. Lowery , R. T. Shroff , K. H. Weiss , C. Springfeld , M. J. Borad , R. K. Ramanathan , L. Goyal , S. Sadeghi , T. Macarulla , A. El‐Khoueiry , R. K. Kelley , I. Borbath , S. P. Choo , D. Y. Oh , P. A. Philip , L. T. Chen , T. Reungwetwattana , E. Van Cutsem , K. H. Yeh , K. Ciombor , R. S. Finn , A. Patel , S. Sen , D. Porter , R. Isaacs , A. X. Zhu , G. K. Abou‐Alfa , T. Bekaii‐Saab , J. Clin. Oncol. 2018, 36, 276. 29182496 10.1200/JCO.2017.75.5009 PMC6075847

[45] X. Liu , E. Krawczyk , F. A. Suprynowicz , N. Palechor‐Ceron , H. Yuan , A. Dakic , V. Simic , Y. L. Zheng , P. Sripadhan , C. Chen , J. Lu , T. W. Hou , S. Choudhury , B. Kallakury , D. G. Tang , T. Darling , R. Thangapazham , O. Timofeeva , A. Dritschilo , S. H. Randell , C. Albanese , S. Agarwal , R. Schlegel , Nat. Protoc. 2017, 12, 439. 28125105 10.1038/nprot.2016.174 PMC6195120

[46] R. H. Wang , C. Li , C. X. Deng , Int. J. Biol. Sci. 2010, 6, 682. 21103071 10.7150/ijbs.6.682 PMC2990071

[47] M. Pertea , D. Kim , G. M. Pertea , J. T. Leek , S. L. Salzberg , Nat. Protoc. 2016, 11, 1650. 27560171 10.1038/nprot.2016.095 PMC5032908

[48] M. I. Love , W. Huber , S. Anders , Genome Biol. 2014, 15, 550. 25516281 10.1186/s13059-014-0550-8 PMC4302049

[49] G. C. Yu , L. G. Wang , Y. Y. Han , Q. Y. He , OMICS 2012, 16, 284. 22455463 10.1089/omi.2011.0118 PMC3339379

[50] R. Sivakumar , M. Chan , J. S. Shin , N. Nishida‐Aoki , H. L. Kenerson , O. Elemento , H. Beltran , R. Yeung , T. S. Gujral , Oncoimmunology 2019, 8, e1670019. 31741771 10.1080/2162402X.2019.1670019 PMC6844320

[51] J. Ferlay , M. Colombet , I. Sorejomataram , D. M. Parkin , M. Piñeros , A. Znaor , F. Bray , J. Int. Cancer 2021, 149, 778.

