# Activation of FGFR2 Signaling Suppresses BRCA1 and Drives Triple‐Negative Mammary Tumorigenesis That is Sensitive to Immunotherapy

**DOI:** 10.1002/advs.202307935

**Published:** 2023-12-28

**Authors:** Josh Haipeng Lei, Mi‐Hye Lee, Kai Miao, Zebin Huang, Zhicheng Yao, Aiping Zhang, Jun Xu, Ming Zhao, Zenan Huang, Xin Zhang, Si Chen, NG Jiaying, Yuzhao Feng, Fuqiang Xing, Ping Chen, Heng Sun, Qiang Chen, Tingxiu Xiang, Lin Chen, Xiaoling Xu, Chu‐Xia Deng

*Adv. Sci*. **2021**, *8*, 2100974

https://doi.org/10.1002/advs.202100974

In the original published article, there are errors in **Figures** **3**, **4**, **5**, and **S3**. The correct figures are shown below. These mistakes were made during the process when organizing these figures. These errors were made unintentionally and do not affect the results or conclusions of this article. The authors apologize for any inconvenience this may have caused.

**Figure 3:**
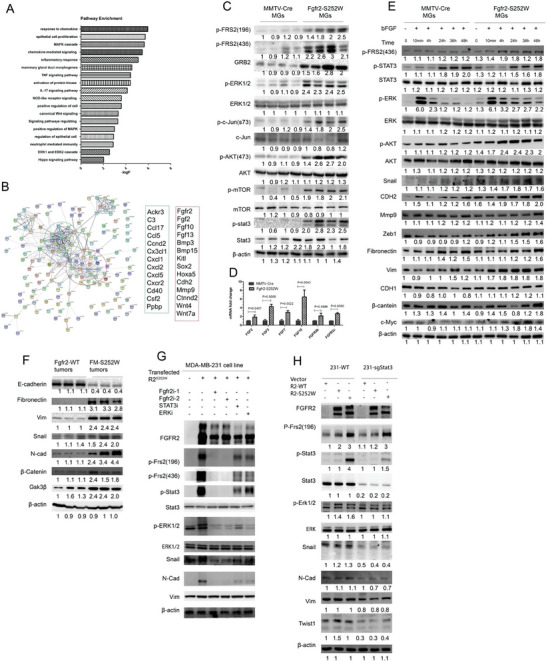
Fgfr2 activation promotes EMT mediated by STAT3‐MAPK signaling. A,B) RNA‐Seq showing differentially expressed genes between *Fgfr2‐WT* and *Fgfr2‐S252W* mammary glands illustrated by pathway enrichment A) and string analysis B). C) Representative Western blot analysis performed on whole‐cell lysates from *Fgfr2‐WT* and *Fgfr2‐S252W* mammary glands to assess the expression of GRB2, FRS2 (Tyr196), FRS2 (Tyr436), ERK1/2 (Thr202/Tyr204), STAT3 (Tyr705), AKT (Ser473), c‐Jun (Ser73), mTOR (Ser2448), and actin. D) Gene profile (FGFs) of Fgfr2‐mediated alterations in the *Fgfr2‐WT* and Fgfr2‐S252W mammary glands analyzed by using real‐time RT‐PCR. Total RNA isolated from 6‐month‐old control and *Fgfr2‐S252W* mice (*n* = 3) was used. E) Fgfr2‐WT and Fgfr2‐S252W mammary gland cells were serum‐starved for 12 h, treated with bFGF for the indicated times, and analyzed for FRS2, ERK1/2, AKT, and EMT markers by WB with antibodies as indicated. F) Representative WB analysis showing expression of EMT marker genes in Fgfr2‐WT and Fgfr2‐S252W mammary gland tumors. G) Representative WB analysis showing alteration of EMT markers with inhibitors to block FGFR2, STAT3, or ERK signaling in MDA‐MB‐231 cells. H) Representative WB analysis showing effects of transfected FGFR2‐WT and FGFR2‐S252W into STAT3 knockout MDA‐MB‐231 cells. Data represent the mean ± SEM and are representative of three independent experiments. *p* values were determined by ANOVA with Tukey's multiple comparison test D). Statistical analysis was carried out using GraphPad Prism 7 Software. *p < 0.05, **p < 0.01, ***p < 0.001, ****p < 0.0001.

**Figure 4:**
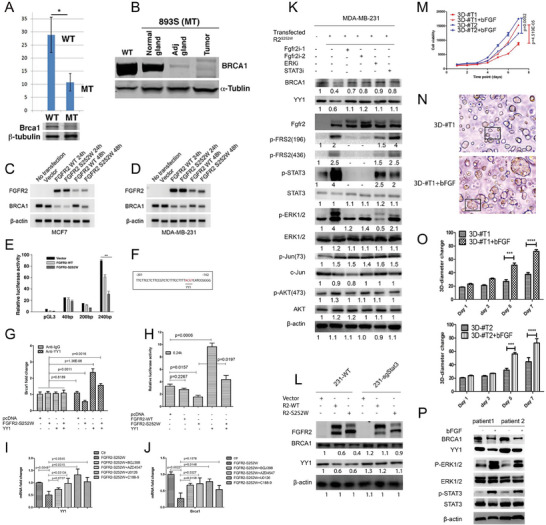
FGFR2 activation negatively regulates BRCA1 by suppressing transcription factor YY1 mediated by the FRS2*𝛼*/STAT3/MAPK pathways. A,B) Expression of Brca1 revealed by RT‐PCR and WB in *Fgfr2‐WT* and *Fgfr2‐S252W* tumors. C,D) BRCA1 mRNA level expression after transfected FGFR2‐WT and FGFR2‐S252W in MDA‐MB‐231 andMCF7 cells revealed by RT‐PCR. E) Luciferase activities of BRCA1 reporter constructs inMDA‐MB‐231 cells. Fold change in luciferase activity of the BRCA1 promoter after transfection of FGFR2‐WT and FGFR2‐S252W. F) Transfection factor YY1 binding site. G‐H) ChIP assay showing that FGFR2‐S252W can compete with YY1 binds to the BRCA1 promoter inMDA‐MB‐231 cells transiently transfected FGFR2‐S252W and co‐transfected with YY1. I,J) FGFR2 mainly though FGFR2/FRS2/MAPK signaling to regulate YY1 and BRCA1. The functionality of FGFR2 in MDAMB‐231 cells as revealed by transfection of FGFR2 overexpression vector or empty vector for 24 h, then treated with multiple inhibitors for another 24 h. Expression of YY1(I) and BRCA1(J) were measured by RT‐PCR. K) FGFR2 suppress BRCA1 and YY1 were rescued by inhibit FGFR/FRS2/STAT3/MAPK signaling evaluated by immunoblotting. Actin was used as a loading control. L) Immunoblotting assay evaluating the expression of YY1/BRCA1 in STAT3‐deficiant cells after transfecting with FGFR2‐WT and FGFR2‐S252W. M–P) Growth curves of two organoid lines (3D‐#T1 and 3D‐#T2) with/without bFGF treatment measured by MTT continuously up to 7 days M), sizes of the organoids N,O) measured by using image J, and expression of some downstream signaling revealed by western blot (P). Data represent the mean ± SEM and are representative of three independent experiments. *p* values were determined by ANOVA with Tukey's multiple comparison test E, G, H, I, J, M, and O). Statistical analysis was carried out using GraphPad Prism 7 Software. *p < 0.05, **p < 0.01, ***p < 0.001, ****p < 0.0001.

**Figure 5:**
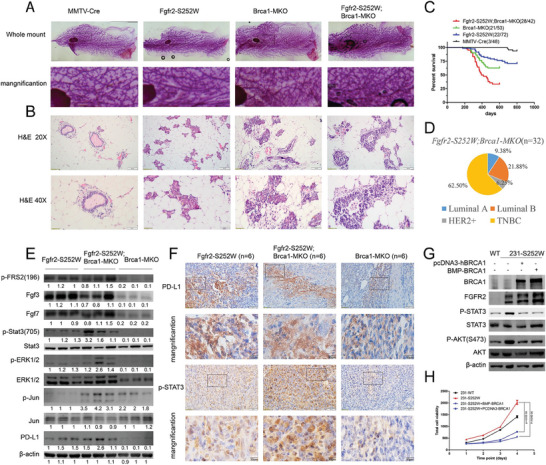
Cooperation between Fgfr2 activation and Brca1 deficiency accelerates mammary tumorigenesis. A,B) Defatted and carmine‐red stained wholemount mammary glands A) and H&E staining B) with indicated genotype. C) Percentage of mammary tumor‐free mice with indicated genotype (*n* = number of mice). D) Percentage of mammary tumors molecule subtypes with indicated genotype (*n* = number of tumor). E) Representative WB analysis performed on whole‐cell lysates from tumors indicated. F) IHC staining against PD‐L1 and pSTAT3 in tumors with genotype indicated. G) Western blots using various antibodies for the effect of ectopic expression of BRCA1 on 231‐S252W cells. H) Two BRCA1 expression plasmids were transfected into 231‐WT and 231‐S252W cells. 24 h later, 3000 cells were seeded into each well of 96‐wells plate. Cells were monitored for four consecutive days and cell proliferation measured and quantified by Alamar blue. Data represent the mean ± SEM and are representative of three independent experiments. *p* values were determined by ANOVA with Tukey's multiple comparison test (H). Statistical analysis was carried out using GraphPad Prism 7 Software. *p < 0.05, **p < 0.01, ***p < 0.001, ****p < 0.0001.

**Figure S3:**
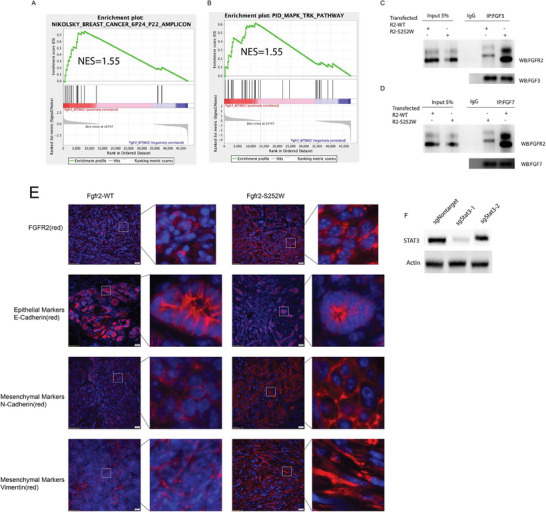
Fgfr2 activation promotes EMT mediated by STAT3‐MAPK signaling: A‐B) GSEA analysis of Fgfr2‐WT and Fgfr2‐S252W mammary glands. The Gene Set Enrichment Analysis (GSEA) program revealed high levels of breast cancer pathway (A) and MAPK signaling (B). C‐D) Interaction of FGFR2 with FGFs. Interaction of FGFR2‐WT and FGFR2‐S252W with FGF3 (C) and FGF7 (D) revealed by co‐IP to identify protein–protein interaction. We transfected FGFR2‐WT and FGFR2‐S252W plasmids into MDA‐MB‐231 cell line followed by co‐IP using anti‐FGF3 or FGF7 antibody. Western blot on the inputs showed comparable levels of FGFR2‐WT and FGFR2‐S252W in the transfected cells, whereas Co‐IP with endogenous FGF3 or FGF7 detected much more abounded FGFR2‐S252W than FGFR2‐ WT protein, indicating much increased FGFR2‐S252W to ligand binding than FGFR2‐WT. E) IF staining against EMT markers (CDH1, CDH2, VIM) with indicated genotype. F) Representative Immunoblotting showing STAT3 knockout by CRISPR‐Cas9 system.

