# Supplementary material for: Activation of FGFR2 Signaling Suppresses BRCA1 and Drives Triple‐Negative Mammary Tumorigenesis That is Sensitive to Immunotherapy
Source: Adv Sci (Weinh). 2021 Sep 13;8(21):2100974. doi: 10.1002/advs.202100974 (PMC8564435; doi:10.1002/advs.202100974)
Supplement: Supplementary file 1 — Supporting Information [file ADVS-8-2100974-s001.pdf]

© 2021 Wiley-VCH GmbH

ADVANCED SCIENCE
Open Access

Supporting Information

for *Adv. Sci.,* DOI: 10.1002/advs.202100974

Activation of FGFR2 Signaling Suppresses BRCA1 and Drives Triple-Negative Mammary Tumorigenesis that Is Sensitive to Immunotherapy

*Josh Haipeng Lei, Mi-Hye Lee, Kai Miao, Zebin Huang, Zhicheng Yao, Aiping Zhang, Xu Jun, Ming Zhao, Zenan Huang, Xin Zhang, Si Chen, NG Jiaying, Yuzhao Feng, Fuqiang Xing, Ping Chen, Heng Sun, Qiang Chen, Lin Chen, Xiaoling Xu, and Chu-Xia Deng**

Supporting Information

Activation of FGFR2 Signaling Suppresses BRCA1 and Drives Triple-Negative Mammary Tumorigenesis that Is Sensitive to Immunotherapy

*Josh Haipeng Lei[1,2,3], Mi-Hye Lee[4], Kai Miao[1,2,3], Zebin Huang[1], Zhicheng Yao[5], Aiping Zhang[1,2], Xu Jun[1,2], Ming Zhao[1,2], Zenan Huang[6], Xin Zhang[1,2], Si Chen[1,2], NG Jiaying[1], Yuzhao Feng[1], Fuqiang Xing[1,2], Ping Chen[1,2], Heng Sun[1,2,3], Qiang Chen[1,2,3], Lin Chen[7], Xiaoling Xu[1,2,3], and Chu-Xia Deng[1,2,3]**

[1]Cancer Center, Faculty of Health Sciences, University of Macau, Macau SAR, China.

[2]Institute of Translational Medicine, Faculty of Health Sciences, University of Macau, Macau SAR, China

[3]Frontier Science Centre for Precision Oncology, University of Macau, Taipa, Macau SAR, China

[4]Department of Oncology, Georgetown-Lombardi Comprehensive Cancer Center, Georgetown University, Washington, DC, USA.

[5]Department of General Surgery, The Third Affiliated Hospital of Sun Yat-Sen University, Guangzhou, Guangdong, China.

[6]Department of Thyroid and Breast Surgery, The Third Affiliated Hospital of Sun Yat-Sen University, Guangzhou, Guangdong, China.

[7]Center of Bone Metabolism and Repair, Department of Rehabilitation Medicine, State Key Laboratory of Trauma, Burns and Combined Injury, Trauma Center, Research Institute of Surgery, Daping Hospital, Third Military Medical University, Chongqing, China.

*Corresponding authors:

Chu-Xia Deng: cxdeng@um.edu.mo

Table. 1 primers used for experiments

q-m-Brca1-F1	CAGCAGGAGCCAAATCTATAAGC
q-m-Brca1-R1	CTTCACTGCTACCACAACTATCG
q-m-YY1-F1	CGACCAGGCCAAAAAGACAAG
q-m-YY1-R1	CACATCGCAGATGCTGCATT
Q-h-YY1-F1	GGAGGAATACCTGGCATTGA
Q-h-YY1-R1	GGTT GTTTTTGGCCTTAGCA
Q-h-BRCA1-F1	GAAGA AACCACCAAGGTCCA
Q-h-BRCA1-R1	GGGATCTGGG GTATCAGGTA
q-mFGF2 F1	ACCAGGCCACTTCAAGGAC
q-mFGF2 R1	GCCGTCCATCTTCCTTCATA
q-mFGF3 F1	GCAAGCTCTACTGCGCTACC
q-mFGF3 R1	TGCGTTGTAGTGATCCGAAG
q-mFGF7 F1	GAACAAAAGTCAAGGAGCAACC
q-mFGF7 R1	GTCATGGGCCTCCTCCTATT
q-mFGF10 F1	GAGAAGAACGGCAAGGTCAG
q-mFGF10 R1	CTCTCCTGGGAGCTCCTTTT
q-mFgfr2 IIIb F1	AAGGTTTACAGCGATGCCCA
q-mFgfr2 IIIb R1	AGAGCCAGCACTTCTGCATT
q-mFgfr2 IIIc F1	GTGTTAACACCACGGACAAA
q-mFgfr2 IIIc R1	TGGCAGAACTGTCAACCATG
q-mTGFβ1 -F1	AAGTTGGCATGGTAGCCCTT
q-mTGFβ1 -R1	GCCCTGGATACCAACTATTGC
sgh-STAT3-F1	AGATTGCCCGGATTGTGGCC
sgh-STAT3-R1	GGCCACAATCCGGGCAATCT
sgh-STAT3-F2	AGAGAACATTCGACTCTTGC
sgh-STAT3-R2	GCAAGAGTCGAATGTTCTCT
BRCA1 promoter-F1	TTTATGGCAAACTCAGGTAG
BRCA1 promoter-R1	CACGCCAGTACCCCAGAGCA

Table. 2 Antibodies used for experiments

Item Description	Source	Model / Cat. #	IHC/IF	WB
Anti-Estrogen Receptor alpha antibody	Abcam	ab3575	1:200	
Anti-Progesterone Receptor antibody	Abcam	ab101688	1:500	
HER2/ErbB2 (29D8) Rabbit mAb #2165	CST	#2165	1:200	
Anti-FGFR2	Abcam	ab10648	1:500	1:1000
Anti-FRS2	Abcam	ab10425		1:1000
Anti-mTOR (phospho S2448) antibody	Abcam	ab109268		1:1000
Anti-mTOR antibody	Abcam	ab32028		1:1000
Anti-N Cadherin	Abcam	ab76057	1:500	1:1000
BRCA1 Antibody (C-20)*	Santa Cruz	sc-642	1:50	1:100 Human/Mus
BRCA1 Antibody (D-9)*	Santa Cruz	sc-6954	1:50	1:100 Human
BRCA1 Antibody(287.17)*	Santa Cruz	sc-135732	-	1:100 Mus
YY1(H10)	Santa Cruz	sc-7341	1:100	1:500
CD8α (D8A8Y) Rabbit mAb	CST	#85336	1:400	
E-Cadherin (4A2)	CST	#14472	1:250	1:1000
FGF Receptor 2 (D4L2V) Rabbit mAb	CST	#23328S		1:1000
FGF-3 Antibody (A-5)	Santa Cruz	sc-374608		1:100
FGF-7 Antibody (F-9)	Santa Cruz	sc-365440		1:100
GSK-3β (D5C5Z)	CST	#12456		1:1000
H-Ras Antibody (259)	Santa Cruz	sc-35		1:100
Keratin 8/18 (C51)	CST	#4546	1:200	
Keratin 14	Abcam	Ab5105	1:200	
p44/42 MAPK (Erk1/2)	CST	#4695S		1:1000
PD-L1 (E1L3N®) XP® Rabbit mAb	CST	#13684S	1:200	1:1000
Phospho-c-Jun (Ser73)	CST	#3270S		1:1000
Phospho-FRS2-α (Tyr196)	CST	#3864L		1:1000
Phospho-FRS2-α (Tyr436)	CST	#3861S		1:1000
Phospho-p44/42 MAPK (Erk1/2)	CST	#4370L		1:1000
Phospho-Stat3 (Tyr705)	CST	#9145S		1:1000
Stat3 (124H6)	CST	39139S		1:1000
twist1	Santa Cruz	sc-81417		1:200
Vimentin (D21H3)	CST	#5741		1:1000

γ-H2AX	CST	#2577		1:1000

* Three antibodies to BRCA1 were used in a comparative way for detecting correct band of BRCA1 in Western blot.

Table 3 Antibodies used for experiments

Item Description	Source	Model / Cat. #	Flow
CD3e Monoclonal Antibody (145-2C11), Alexa Fluor 488	ThermoFisher Scientific	Cat# 53-0031-82; RRID:AB_469889	1:1000
CD8a Monoclonal Antibody (53-6.7), PerCP-eFluor 710	ThermoFisher Scientific	Cat# 46-0081-82; RRID:AB_1834433	1:1000
CD44 Monoclonal Antibody (IM7), PE	ThermoFisher Scientific	Cat# 12-0441-82; RRID:AB_465664	1:1000
CD62L (L-Selectin) Monoclonal Antibody (MEL14), eFluor 450	ThermoFisher Scientific	Cat# 48-0621-82; RRID:AB_1963590	1:1000
F4/80 Monoclonal Antibody (BM8), eFluor 450	ThermoFisher Scientific	Cat# 14-4801-82; RRID:AB_1548747	1:1000
CD206 (MMR) Monoclonal Antibody (MR6F3), APC	ThermoFisher Scientific	Cat # 17-2061-82; RRID AB_2637420	1:1000

Supporting Information

Activation of FGFR2 Signaling Suppresses BRCA1 and Drives Triple-Negative Mammary Tumorigenesis that Is Sensitive to Immunotherapy

*Josh Haipeng Lei[1,2,3], Mi-Hye Lee[4], Kai Miao[1,2,3], Zebin Huang[1], Zhicheng Yao[5], Aiping Zhang[1,2], Xu Jun[1,2], Ming Zhao[1,2], Zenan Huang[6], Xin Zhang[1,2], Si Chen[1,2], NG Jiaying[1], Yuzhao Feng[1], Fuqiang Xing[1,2], Ping Chen[1,2], Heng Sun[1,2,3], Qiang Chen[1,2,3], Tingxiu Xiang[7], Lin Chen[8], Xiaoling Xu[1,2,3], and Chu-Xia Deng[1,2,3]**

[1]Cancer Center, Faculty of Health Sciences, University of Macau, Macau SAR, China

[2]Institute of Translational Medicine, Faculty of Health Sciences, University of Macau, Macau SAR, China

[3]MOE Frontier Science Centre for Precision Oncology, University of Macau, Taipa, Macau SAR, China

[4]Department of Oncology, Georgetown-Lombardi Comprehensive Cancer Center, Georgetown University, Washington, DC, USA

[5]Department of General Surgery, The Third Affiliated Hospital of Sun Yat-Sen University, Guangzhou, Guangdong, China

[6]Department of Thyroid and Breast Surgery, The Third Affiliated Hospital of Sun Yat-Sen University, Guangzhou, Guangdong, China

[7]Oncology Laboratory, The First Affiliated hospital of Chongqing Medical University, Chongqing, China

[8]Center of Bone Metabolism and Repair, Department of Rehabilitation Medicine, State Key Laboratory of Trauma, Burns and Combined Injury, Trauma Center, Research Institute of Surgery, Daping Hospital, Third Military Medical University, Chongqing, China

*Corresponding author: Chu-Xia Deng: cxdeng@um.edu.mo

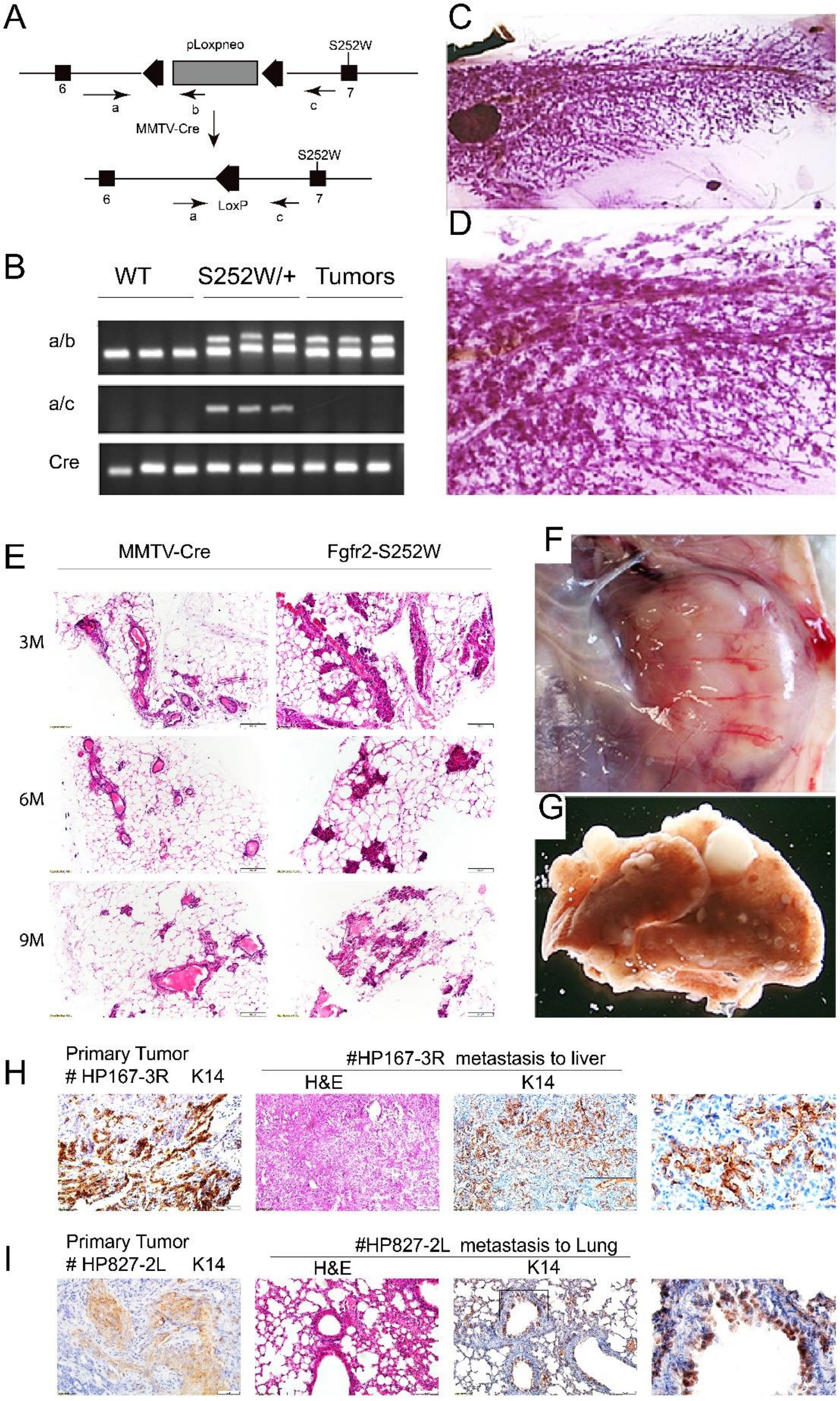
A
pLoxpneo
S252W
6
a
b
c
7
MMTV-Cre
S252W
6
LoxP
a
c
7
B
WT
S252W/+
Tumors
a/b
a/c
Cre
C
D
E
MMTV-Cre
Fgfr2-S252W
3M
6M
9M
F
G
H
Primary Tumor
HP167-3R K14
#HP167-3R metastasis to liver
H&E
K14
I
Primary Tumor
HP827-2L K14
#HP827-2L metastasis to Lung
H&E
K14

Figure S1. *Fgfr2 activation enhances mammary branch morphogenesis and promotes mammary tumorigenesis*

A-B) Structure of ploxPneo-Fgfr2 transgene before and after deletion of ploxPneo. Removal of the ploxPneo gene by crossing with an *MMTV-Cre* transgenic mouse. C-E) Defatted and carmine-red stained whole-mount of *Fgfr2-S252W* (C-D) and H&E images of the fourth abdominal mammary glands from representative *Fgfr2-WT* (*MMTV-Cre*) and *Fgfr2-S252W* mice at different stages of development. H&E sections were prepared from mice at 3 months (3M), 6 months (6M) and 9 months (9M). F-G) *Fgfr2-S252W* mice develop tumour (F) and metastasis to lymph nodules (G) . H-I) Analysis of liver and lung metastasis using mammary cell specific marker K14. H) K14 expression in the primary mammary tumor developed in the 3rd right mammary gland of HP-167 mouse (HP167-3R), and in the liver metastasis. I) K14 expression in the primary mammary tumor developed in the 2nd left mammary gland of HP-827 mouse (HP827-2L), and in the lung metastasis. The marked areas in A and B were enlarged and placed on the right.

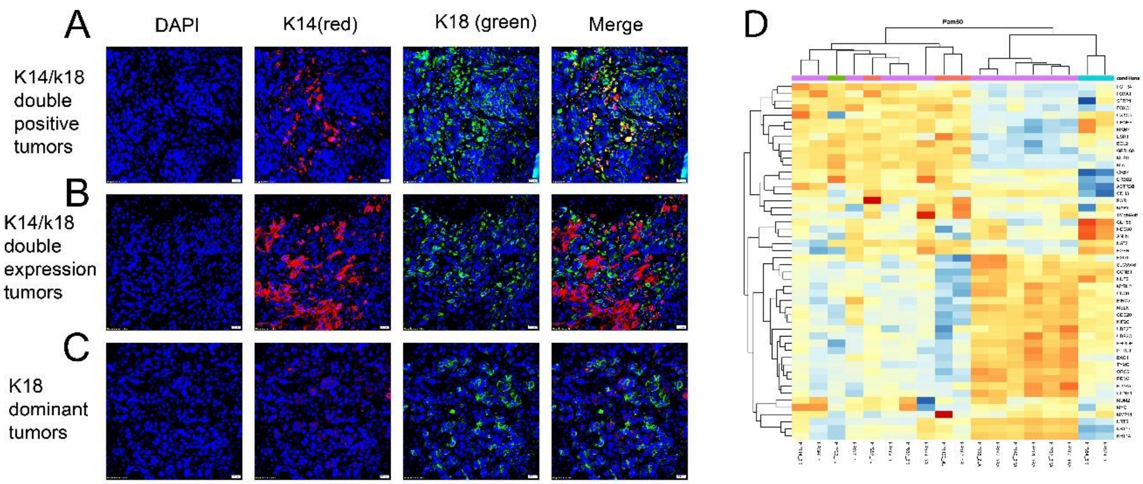

Figure S2. ***Fgfr2 activation promotes the development of triple-negative mammary tumors***

A-C) Immunofluorescence (IF) on paraffin sections using K18 (green) and K14 (red) antibodies. DAPI stains the nuclei. K18 and K14 double positive (A), K18 and K14 double expression (B) and K18 dominant expression (C) in *Fgfr2-S252W* mammary tumors. D) Heatmap clustering according to PAM50 from *Fgfr2-S252W* tumours by RNA-seq..

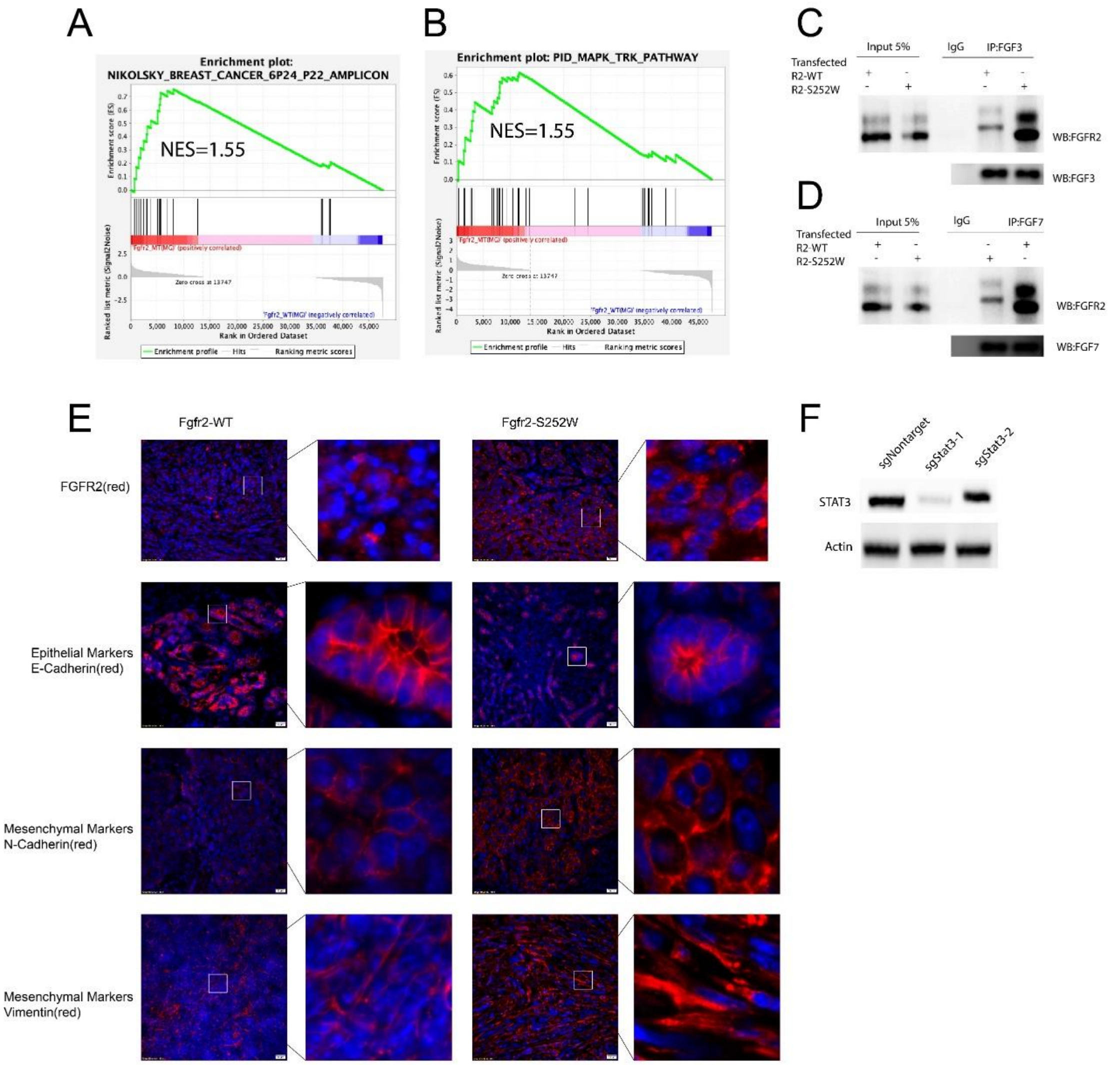
A
Enrichment plot:
NIKOLSKY_BREAST_CANCER_6P24_P22_AMPLICON
NES=1.55
B
Enrichment plot: PID_MAPK_TRK_PATHWAY
NES=1.55
C
Input 5%
IgG
IP:FGF3
Transfected
R2-WT
R2-S252W
WB:FGFR2
WB:FGF3
D
Input 5%
IgG
IP:FGF7
Transfected
R2-WT
R2-S252W
WB:FGFR2
WB:FGF7
E
Fgfr2-WT
Fgfr2-S252W
FGFR2(red)
Epithelial Markers
E-Cadherin(red)
Mesenchymal Markers
N-Cadherin(red)
Mesenchymal Markers
Vimentin(red)
F
sgNontarget
sgStat3-1
sgStat3-2
STAT3
Actin

Figure 3. ***Fgfr2 activation promotes EMT mediated by STAT3-MAPK signaling***

A-B) GSEA analysis of *Fgfr2-WT* and *Fgfr2-S252W* mammary glands. The Gene Set Enrichment Analysis (GSEA) program revealed high levels of breast cancer pathway (A) and MAPK signaling (B). C-D) Interaction of FGFR2 with FGFs. Interaction of FGFR2-WT and FGFR2-S252W with FGF3 (C) and FGF7 (D) revealed by co-IP to identify protein–protein interaction. We transfected FGFR2-WT and FGFR2-S252W plasmids into MDA-MB-231 cell line followed by co-IP using anti-FGF3 or FGF7 antibody. Western blot on the inputs showed comparable levels of FGFR2-WT and FGFR2-S252W in the transfected cells, whereas Co-IP with endogenous FGF3 or FGF7 detected much more abounded FGFR2-S252W than FGFR2-WT protein, indicating much increased FGFR2-S252W to ligand binding than FGFR2-WT. E) IF staining against EMT markers (CDH1, CDH2, VIM) with indicated genotype. F) Representative Immunoblotting showing STAT3 knockout by CRISPR-Cas9 system.

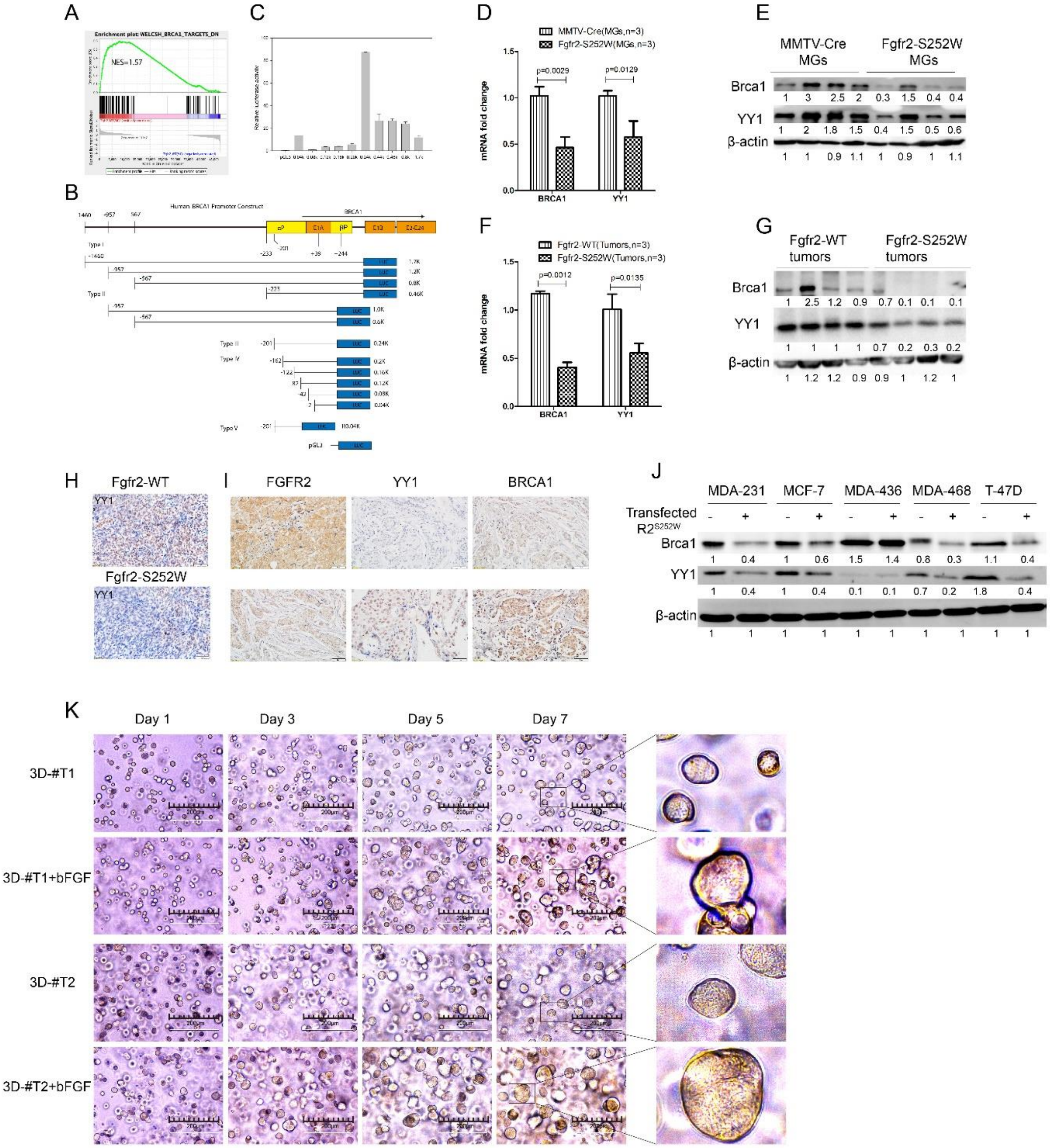
A
Enrichment plot: WELCSH_BRCA1_TARGETS_DN
NES=1.57
C
D
MMTV-Cre(MGs,n=3)
Fgfr2-S252W(MGs,n=3)
p=0.0029
p=0.0129
mRNA fold change
BRCA1
YY1
E
MMTV-Cre MGs
Fgfr2-S252W MGs
Brca1
1 3 2.5 2 0.3 1.5 0.4 0.4
YY1
1 2 1.8 1.5 0.4 1.5 0.5 0.6
β-actin
1 1 0.9 1.1 1 0.9 1 1.1
B
Human BRCA1 Promoter Construct
F
Fgfr2-WT(Tumors,n=3)
Fgfr2-S252W(Tumors,n=3)
p=0.0012
p=0.0135
mRNA fold change
BRCA1
YY1
G
Fgfr2-WT tumors
Fgfr2-S252W tumors
Brca1
1 2.5 1.2 0.9 0.7 0.1 0.1 0.1
YY1
1 1 1 1 0.7 0.2 0.3 0.2
β-actin
1 1.2 1.2 0.9 0.9 1 1.2 1
H
Fgfr2-WT
YY1
Fgfr2-S252W
YY1
I
FGFR2
YY1
BRCA1
J
MDA-231
MCF-7
MDA-436
MDA-468
T-47D
Transfected R2S252W
- + - + - + - + - +
Brca1
1 0.4 1 0.6 1.5 1.4 0.8 0.3 1.1 0.4
YY1
1 0.4 1 0.4 0.1 0.1 0.7 0.2 1.8 0.4
β-actin
1 1 1 1 1 1 1 1 1 1
K
Day 1
Day 3
Day 5
Day 7
3D-#T1
3D-#T1+bFGF
3D-#T2
3D-#T2+bFGF

Figure 4. ***FGFR2 activation negatively regulates BRCA1 by suppressing transcription factor YY1 mediated by the FRS2α/STAT3/MAPK pathways***

A) The Gene Set Enrichment Analysis (GSEA) program revealed Brca1 signaling in *Fgfr2-S252W* tumorigenesis. B-C) Structures of BRCA1 promoter regions (B), and luciferase activities of BRCA1 reporter constructs in MDA-MB-231 cells (C). D-G) Brca1 and YY1 mRNA expression (D-E) in *Fgfr2-WT* or *Fgfr2-S252W* mammary glands (n=3) (D) and *Fgfr2-WT* or *Fgfr2-S252W* tumors (n=3) (E) evaluated by RT-PCR and Western blotting (F-G). H) YY1 immunostaining assays revealed that YY1 displayed decreased expression patterns in the Fgfr2-S252W compared with Fgfr2-WT tumors. Scale bars: 20 µm. I) Representative Immunohistochemical (IHC) staining of FGFR2, YY1 and BRCA1. J) Human TNBC (231, 436, 468) and Non-TNBC (MCF-7, T47D) cells were transfected with or without FGFR2-S252W plasmid incubated for 24 hours. YY1 and BRCA1 were evaluated by Immunoblotting. K) Two organoid lines (3D-#T1 and 3D-#T2) with/without bFGF treatment. Diameters of organoids of 3D-#T1 and 3D-#T2 measured by using image J and Images of these organoids at Day 1, 3, 5 and 7. Data represent the mean ± SEM and are representative of three independent experiments. P values were determined by ANOVA with Tukey's multiple comparison test (D and E). Statistical analysis was carried out using GraphPad Prism 7 Software. *p < 0.05, **p < 0.01, ***p < 0.001, ****p < 0.0001.

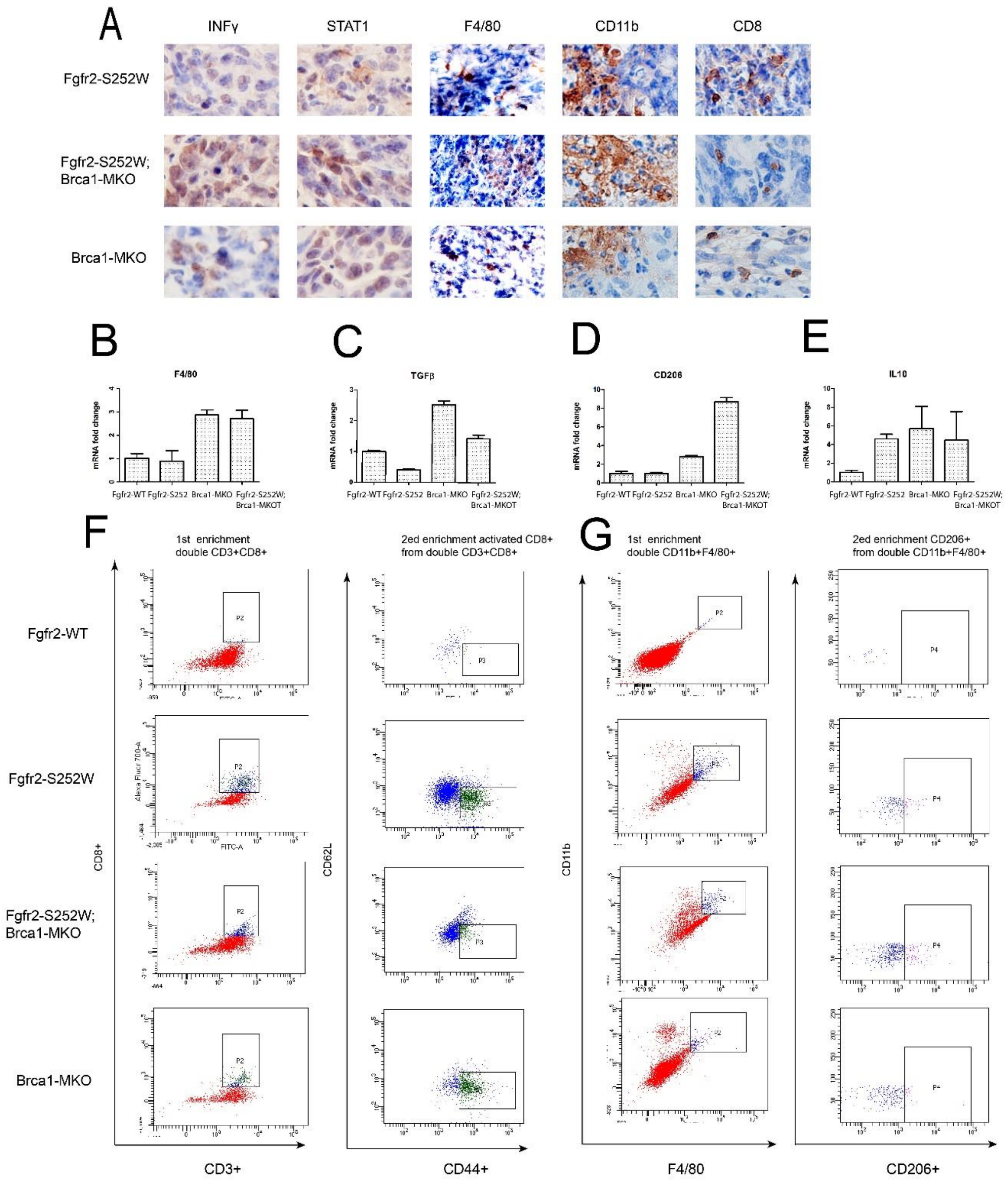

Figure S5. ***PD-L1 expression is promoted by FGFR2-induced STAT3 and ERK activation***

A) IHC staining against IFNγ, STAT1, F4/80, CD11b and CD8 with indicated genotypes. B-E) RT-PCR analysis of inflammatory markers (F4/80, CD206, TGFβ and IL10) in tumor cells with

indicated genotypes. F-G) Flow cytometric analysis of CD3+, CD8+, CD44, F4/80, CD11b, and CD206+ cells in tumor cells with indicated genotypes (n=6).

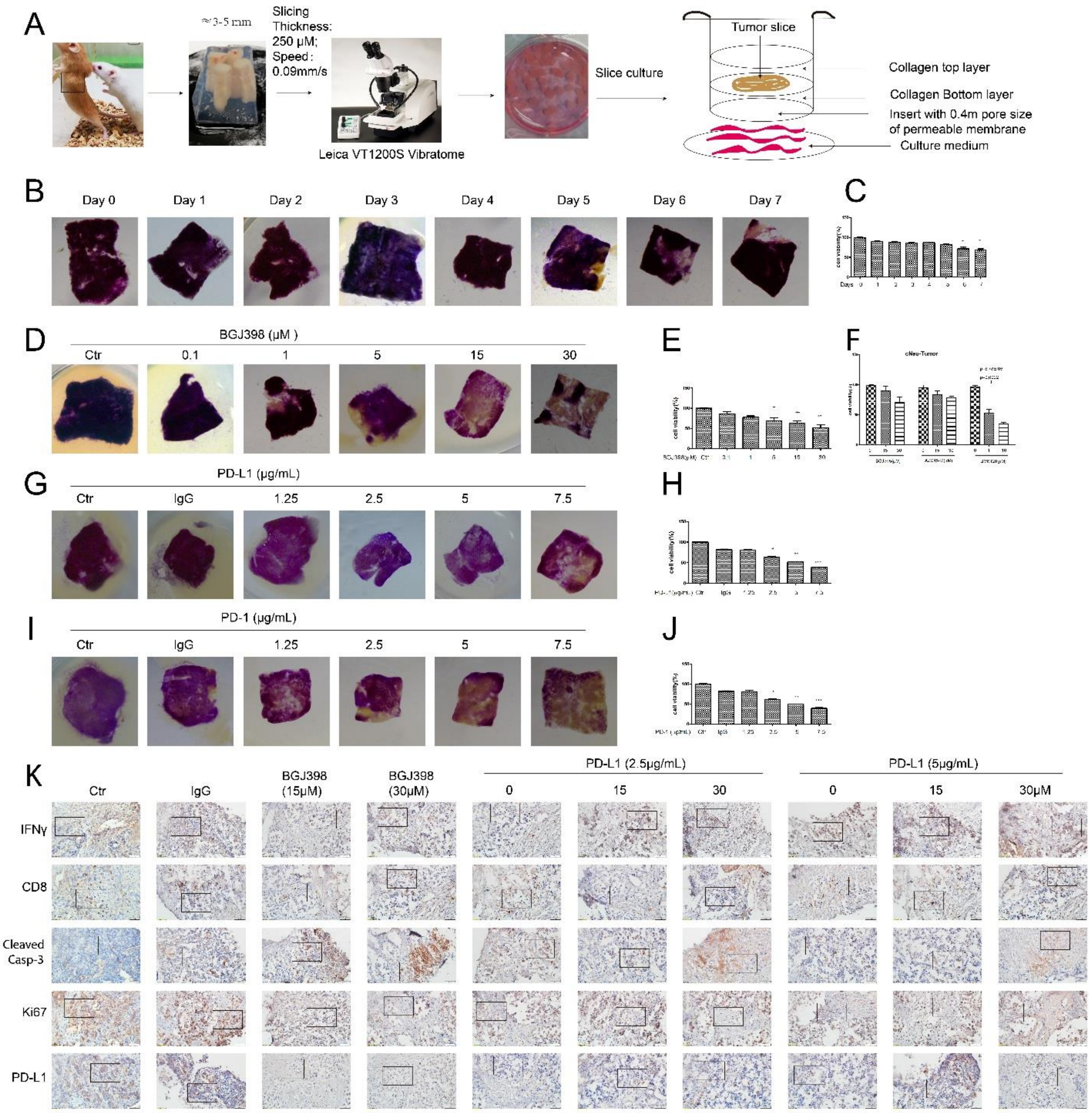

Figure S6. ***Establishment of a tumor slice culture platform for rapid evaluation of the therapeutic efficacy of anticancer drugs***

A) Diagram depicting the workflow of preparation, culturing and analysis of precision-cut tissue slices. B-C) Measurement of cell proliferation of tissue at the processing time of 1, 2, 3, 4, 5, 6 and 7 days using MTT assay. D-E) MTT analysis of tumor slice from *Fgfr2-S252W* tumors with BGJ398 at various concentrations for 4 days. F) MTT analysis of tumor slice from *MMTV-cNeu* with various inhibitor for 4 days. G-J) MTT analysis of tumor slice with anti-PD-1/PD-L1 at various concentrations for 4 days. K) IHC staining against IFNγ, CD8, Cleaved Casp3, Ki67 and PD-L1 with indicated treatment groups. Data represent the mean ± SEM and are representative of three independent experiments. P values were determined by ANOVA with Tukey's multiple comparison test (C, E, F, H, and J). Statistical analysis was carried out using GraphPad Prism 7 Software. *$p < 0.05$, **$p < 0.01$, ***$p < 0.001$, ****$p < 0.0001$.
